# Nonlinear response of vegetation productivity to climate change and human activities in a water-source basin: implications for environmental health and water security

**DOI:** 10.3389/fpubh.2026.1868335

**Published:** 2026-08-26

**Authors:** Hai Liu, Yubing Wang, Tiancheng Wang, Si Chen, Yuhui Bao, Liang Zheng, Min Wang

**Affiliations:** 1 Faculty of Resources and Environmental Science, Hubei University, Wuhan, China; 2 Hubei Provincial Key Laboratory of Regional Development and Environmental Response, Hubei University, Wuhan, China; 3 School of Geographical Science, Hubei University, Wuhan, China; 4 College of Urban and Environmental Sciences, Central China Normal University, Wuhan, China

**Keywords:** anthropogenic activities, climate change, environmental health, gross primary productivity, nonlinear response, water security

## Abstract

**Introduction:**

Gross primary productivity (GPP) is a key component of the global carbon cycle, and its dynamics are influenced by climate conditions and human activities. However, the nonlinear response of GPP to climate change and human activities remains unclear. The Hanjiang River Basin (HJRB), a crucial water-source region of the South-to-North Water Diversion Project in China, is vital to China's ecological security. Changes in vegetation productivity in this water-source basin may affect water conservation, soil erosion control, drought and flood regulation, and the environmental conditions to which downstream populations are exposed. In recent decades, the region has experienced significant climate change, intensified human activities, and notable increases in vegetation greenness. However, previous literature relying on the assumption of stationarity and traditional linear methods has limited capability in detecting nonlinear and gradual vegetation changes.

**Methods:**

The long-term nonlinear trend in GPP in the HJRB was analyzed using ensemble empirical mode decomposition (EEMD), and the nonlinear response relationships among climatic factors, human activities, and GPP were further quantified using the LightGBM machine-learning method.

**Results:**

From 2000 to 2020, the annual growth rate of vegetation productivity in the HJRB was 12.27 g C m^−2^ yr^−1^ (*P* < 0.01). Most vegetated areas exhibited an upward trend in GPP. However, 28.25% and 4.41% of the region experienced transitions from growth to decline and from decline to growth, respectively, primarily in the southern upstream and eastern downstream areas. Drought (25.28%), precipitation (10.75%), and relative humidity (10.69%) were identified as significant factors influencing vegetation productivity trends. Annual precipitation above approximately 1,000 mm was associated with lower SHAP values for the continuous-increase trend, which may reflect combined hydrothermal, radiation, and topographic constraints rather than a direct inhibitory effect of precipitation. Additionally, an increase in SPEI12 was associated with higher SHAP values for the continuous-increase trend.

**Discussion:**

This study elucidates trends and underlying response patterns in vegetation productivity in the HJRB, reveals the driving factors behind regional vegetation productivity changes, and provides environmental evidence for vegetation monitoring, water-source ecosystem management, and climate-related health-risk prevention in the HJRB and similar water-source regions.

## 1 Introduction

Vegetation, as the primary producer in terrestrial ecosystems, plays a crucial role in carbon, water, and energy cycles, thereby maintaining climate balance at both local and global scales (1–3). However, vegetation is highly susceptible to the impacts of climate change, natural disasters, and land use changes. The history of global warming, an increasing frequency of natural disasters, and rapid urbanization have rendered trends in vegetation changes increasingly complex (4–7). The advent and application of remote sensing technology have facilitated the monitoring of terrestrial ecosystems across extensive spatial and temporal scales, enabling the quantification of surface vegetation growth status using various vegetation indices and growth indicators (8–10). Gross primary productivity (GPP), defined as the total amount of carbon compounds produced by plant photosynthesis in a given period, holds significant implications for simulating terrestrial carbon cycling processes and serves as the primary energy source in terrestrial ecosystems (3, 8, 11, 12). The interaction between regional climate change and vegetation change in regulating vegetation growth trends remains uncertain across different scales (2, 13, 14). Thus, investigating the impacts of climate change and human activities on GPP is of paramount importance for addressing future challenges posed by climate change and urbanization.

Accurately monitoring the spatiotemporal evolution and abrupt changes in vegetation is crucial for identifying the driving factors of vegetation change. Traditional linear methods, such as linear regression and correlation analysis, are commonly employed in research on vegetation and climate change (10, 15). However, due to the pronounced seasonality of vegetation and climate change, the linear assumption may oversimplify the evolution of vegetation trends (14, 16). To address this limitation, various signal processing methods have been utilized to decompose vegetation data and identify trend changes, including Empirical Orthogonal Function (EOF) analysis, wavelet analysis, Fourier transform, and Ensemble Empirical Mode Decomposition (EEMD) (17–21). Among them, EEMD is an adaptive decomposition technique that separates data into intrinsic mode functions and trend components at different time scales (22). It is an extension of the Empirical Mode Decomposition (EMD) method (23) and has proven effective in analyzing vegetation growth dynamics and climate change trends (18, 20, 24–26). Simultaneously, there is a complex interplay between vegetation alterations and their driving factors (20, 27). Previous approaches commonly rely on linear assumptions, potentially leading to overestimation or underestimation of the impact of LightGBM, based on the Gradient Boosting Decision Tree (GBDT) framework, which uses decision trees as base learners and can model nonlinear and interactive relationships without imposing a predefined functional form (28). This characteristic makes it suitable for analyzing vegetation responses to climatic and anthropogenic factors, which are often characterized by threshold effect—asymmetric responses and interactions among hydrothermal, topographic, and human activity variables. However, unlike traditional regression models, machine learning models are often criticized for limited interpretability. Therefore, combining LightGBM with SHAP provides a way to quantify the relative contribution of each factor and reveal class-specific nonlinear response patterns. Furthermore, compared with XGBoost (29), LightGBM demonstrated enhanced computational efficiency and has been extensively validated in numerous studies (30–33). This approach has shown superior predictive accuracy compared to alternative machine learning models. Nevertheless, limited research has been conducted to elucidate the intricate interactions between vegetation change trends and their driving factors.

Since the late 20th century, China has undertaken extensive ecological restoration efforts, significantly enhancing vegetation coverage and increasing land carbon sinks (2, 34, 35). The temporal variations in carbon sequestration, dominant mechanisms, and contributions of human activities and climate factors vary across different regions (2, 36–38). The regional hydrothermal conditions exhibit substantial differences due to spatial heterogeneity of climatic conditions, with precipitation and temperature being the primary climatic factors influencing vegetation dynamics. These factors play critical roles in vegetation growth, respiration, and photosynthesis (14, 37). Furthermore, climate variables such as wind speed, atmospheric pressure, potential evapotranspiration, and sunshine duration directly or indirectly influence regional hydrothermal conditions, thereby affecting vegetation growth and coverage. For instance, on the Loess Plateau, in addition to the significant impacts of temperature and precipitation, atmospheric pressure, sunshine duration, and humidity exert strong influences on vegetation growth (10). In the Qilian Mountains region, vegetation growth is significantly affected by temperature, precipitation, wind speed, potential evapotranspiration, and solar radiation (20). Moreover, drought represents a complex climatic phenomenon that profoundly affects vegetation (39). Climatic drought typically arises from prolonged periods of insufficient precipitation coupled with high evaporation rates, resulting in an inadequate water supply. Under drought conditions, soil moisture decreases, restricting plant growth, and leading to leaf dehydration and wilting (40). Drought exhibits complex temporal and spatial characteristics (41), and with global warming, the occurrence and frequency of drought events are increasing (42, 43). These drought conditions significantly impact vegetation vigor, leading to vegetation degradation and posing threats to regional agriculture, water resources, and ecosystems (40, 42, 44–46). Therefore, quantifying the impacts of various climate factors and climate drought on GPP variations is crucial for understanding the relationship between climate change and regional carbon cycling. In addition to climate change, human activities significantly influence vegetation dynamics (47). Urbanization and agricultural development in China have precipitated large-scale changes in land use, including extensive farmland cultivation, urban expansion, and deforestation, which have severely compromised the local ecological environment (2). Nevertheless, substantial progress in vegetation restoration has been achieved through the implementation of various large-scale land conservation and restoration programs, such as the Three North Shelterbelt Development Program and the Grain for Green Program (37, 48–50).

The Hanjiang River Basin (HJRB) in Central China is a core water-source region for the South-to-North Water Diversion Middle Route Project and is home to the Danjiangkou Reservoir. The basin has experienced multiple ecological restoration and engineering activities, including land-cover conversion, afforestation, reservoir-related water conservation, agricultural restructuring, and urban expansion, all of which may influence GPP dynamics. As a key water-source basin, the HJRB provides a representative region for examining how vegetation productivity responds nonlinearly to climatic variability and human activities. Such responses are relevant not only to vegetation monitoring and ecological restoration assessment, but also to water-source conservation and basin-scale ecohydrological management. Changes in vegetation productivity may influence soil and water conservation, sediment retention, non-point source pollution risk, and the regulation of drought-, flood-, and heat-related environmental exposures. Land-use change and vegetation greening can influence GPP through coupled water-carbon processes, and their positive and negative effects may offset each other at the basin scale (51). Therefore, nonlinear changes in GPP in the HJRB can be regarded as environmental-health-relevant ecosystem processes linked to water security and the environmental determinants of health in water-source regions. Most previous GPP trend studies have relied on linear trend analysis, correlation analysis, or regression-based attribution methods. These approaches are useful for detecting monotonic changes and average relationships, but they are less effective at identifying nonlinear trend shifts, threshold responses, and interactions among climatic and anthropogenic factors. In particular, conventional linear models typically assume stationary and additive relationships, whereas vegetation productivity may respond asymmetrically to drought, humidity, radiation, topography, and human disturbance.

The objective of this study was to elucidate the nonlinear variation trends of GPP in the HJRB and to clarify how these trends respond to climatic and human activity factors in a water-source basin. By linking nonlinear changes in vegetation productivity to climate variability and anthropogenic pressures, this study provides environmental evidence for understanding ecosystem-human interactions, water-source security, and climate-related exposure risks. Compared with conventional linear trend analysis and regression-based attribution methods, the EEMD LightGBM SHAP framework enables us to identify nonlinear trend transitions, capture complex interactions among factors, and interpret the nonlinear responses of GPP trend types to climatic and human activity factors. The specific goals of this research were: (1) to conduct a nonlinear analysis of the spatiotemporal characteristics of GPP in the HJRB using EEMD; (2) to quantify the contributions of various factors to the variations in GPP in the HJRB using the LightGBM model; and (3) to analyze and investigate the nonlinear response mechanisms of GPP in the HJRB to diverse influencing factors. This study provides clear insights into the trends and response mechanisms of GPP in the HJRB. The results can inform the formulation of rational water resource management and ecological conservation strategies to adapt to potential intensified climate change and drought risks in the future. This is of great significance for maintaining the ecological environment in the HJRB and the environmental protection of similar regions.

## 2 Materials and methods

### 2.1 Study area

**Figure 1:**
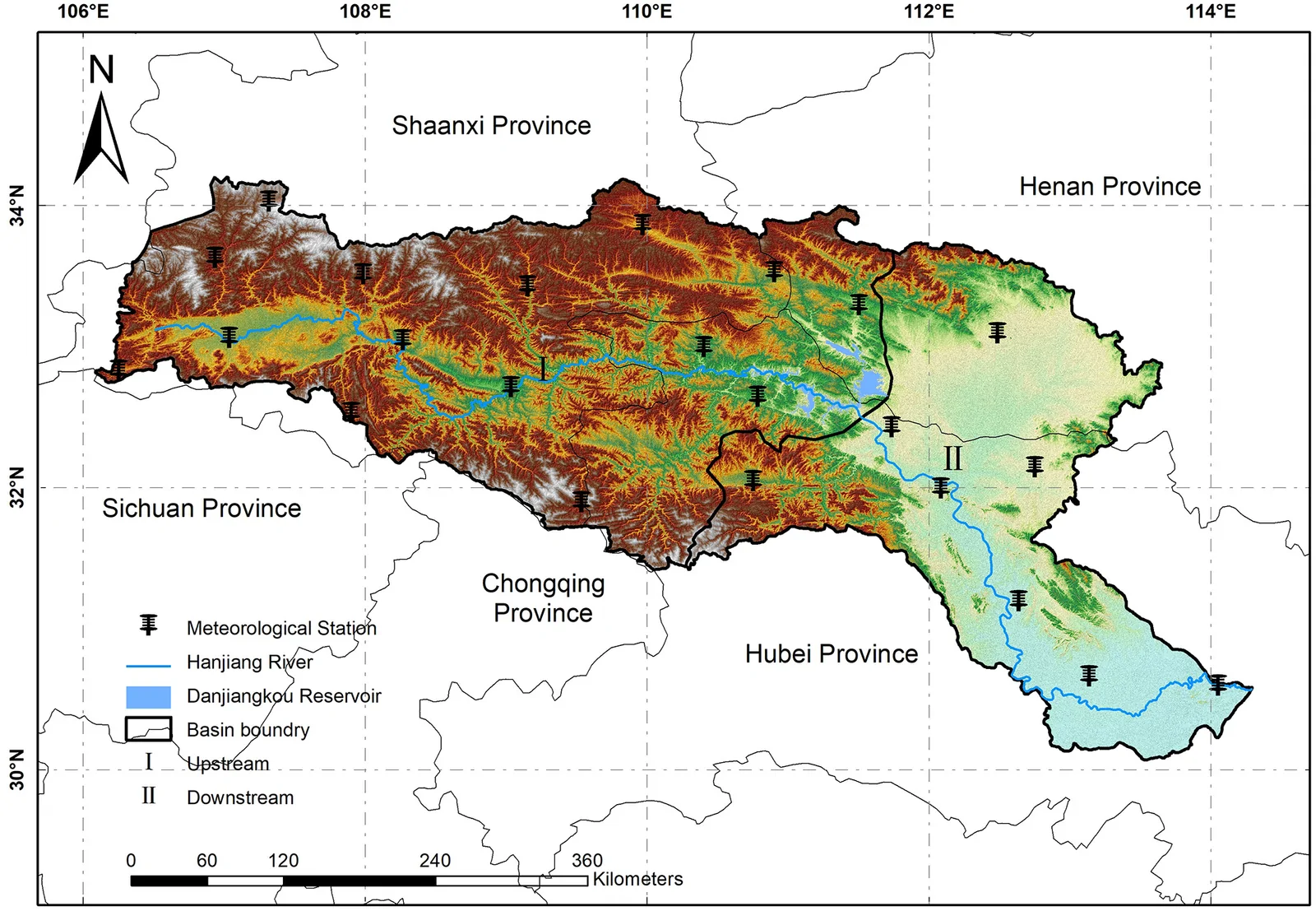
Location of the HJRB.

The HJRB, with a total length of 1,567 km, flows through Shaanxi and Hubei provinces and encompasses a basin area of approximately 15.9 × 104 km^2^, positioning it as one of the largest tributaries of the Yangtze River (Figure 1). The HJRB is characterized by a subtropical monsoon climate, with abundant and diverse water resources. The annual precipitation ranges from 700 mm to 1,100 mm, with 70%−80% occurring during the rainy season from May to September (52). The upper reaches of the HJRB traverse four provinces: Qinghai, Gansu, Sichuan, and Shaanxi. This region is characterized by mountainous and hilly terrain, narrow river valleys, numerous gorges, and treacherous rapids, forming a complex and intricate river system. The lower reaches of the main stem primarily flow through Hubei Province, forming a floodplain with an intricate network of rivers and lakes that harbor abundant water resources and diverse wetland ecosystems. Moreover, the lower reaches of the HJRB serve as the core region of the HJRB Economic Belt, hosting vital transportation hubs and densely populated areas and exerting significant influence on the socioeconomic development of Central China.

### 2.2 Data sources and preprocessing

#### 2.2.1 Gross primary productivity

The GPP data utilized in this research were sourced from the MOD17A2HGF version 6 product of the Moderate Resolution Imaging Spectroradiometer (MODIS) (https://lpdaac.usgs.gov/products/mod17a2hgfv006/). This dataset computes 8-day composite GPP values predicated on the radiation use efficiency paradigm. The temporal coverage extends from January 2000 to December 2020, with a spatial resolution of 500 m. MODIS GPP data have been extensively used in both global and regional studies focused on carbon cycling and vegetation dynamics. In this study, the dataset underwent several preprocessing steps, including projection, cropping, and data format conversion, to derive annual GPP data specific to the HJRB.

#### 2.2.2 Meteorological data

The meteorological data utilized in this research were sourced from the National Meteorological Science Data Center (http://data.cma.cn/). The dataset includes station-based meteorological factors such as precipitation (PRE), temperature (TEM), sunshine duration (SSD), relative humidity (RHU), and wind speed (WS). The temporal coverage spans from 2000 to 2020. Monthly and annual values of these climate factors were computed by aggregating station data. Because the EEMD trend classification was based on annual GPP time series, the climatic variables were also aggregated into annual-scale indicators to ensure temporal consistency with the response variable. Therefore, these variables mainly represent annual background climatic conditions and long-term climatic trends rather than short-term extreme climatic disturbances. Furthermore, the standardized precipitation evapotranspiration index (SPEI) has been observed to be more sensitive in monitoring vegetation productivity trends (53). Consequently, this study used standardized evapotranspiration indices (SPEI1, SPEI3, and SPEI12), which were first calculated at a monthly resolution to represent short-term, seasonal, and long-term drought conditions, respectively. Because the EEMD trend types of GPP were derived from annual GPP time series, the monthly SPEI series were aggregated to annual-scale drought indicators before being used in the LightGBM model. Specifically, SPEI12 was used to represent long-term annual drought stress, whereas SPEI1 and SPEI3 were interpreted as annual summaries of short-term and seasonal drought anomalies rather than direct monthly or seasonal drought events. The SPEI calculations were performed using R 4.1.2, with the potential evapotranspiration (PET) estimated using the Penman-Monteith method (54, 55). To ensure data consistency, the spatial resolution of the climate factors was interpolated to match that of the GPP data using ANUSPLINE (56). The climate variables employed in this study are summarized in Table 1.

**Table 1:** Description of the 10 climatic indices.

Climatic index	ID	Definition	Unit
Precipitation	PRE	Yearly total amount of precipitation	mm
Temperature	TEM	Yearly average value of daily temperature	°C
Potential evapotranspiration	PET	Yearly total amount of potential evapotranspiration	mm
Air pressure	PRS	Yearly average value of daily air pressure	kPa
Sunshine duration	SSD	Yearly average value of daily sunshine duration	Hours
Wind speed	WS	Yearly average value of daily wind speed	m/s
Relative humidity	RHU	Yearly average value of daily relative humidity	%
SPEI1	SPEI1	Annual summary of monthly SPEI1, representing short-term drought anomalies	
SPEI3	SPEI3	Annual summary of monthly SPEI3, representing seasonal drought anomalies	
SPEI12	SPEI12	Annual scale SPEI12, representing long-term drought stress	

**Figure 2:**
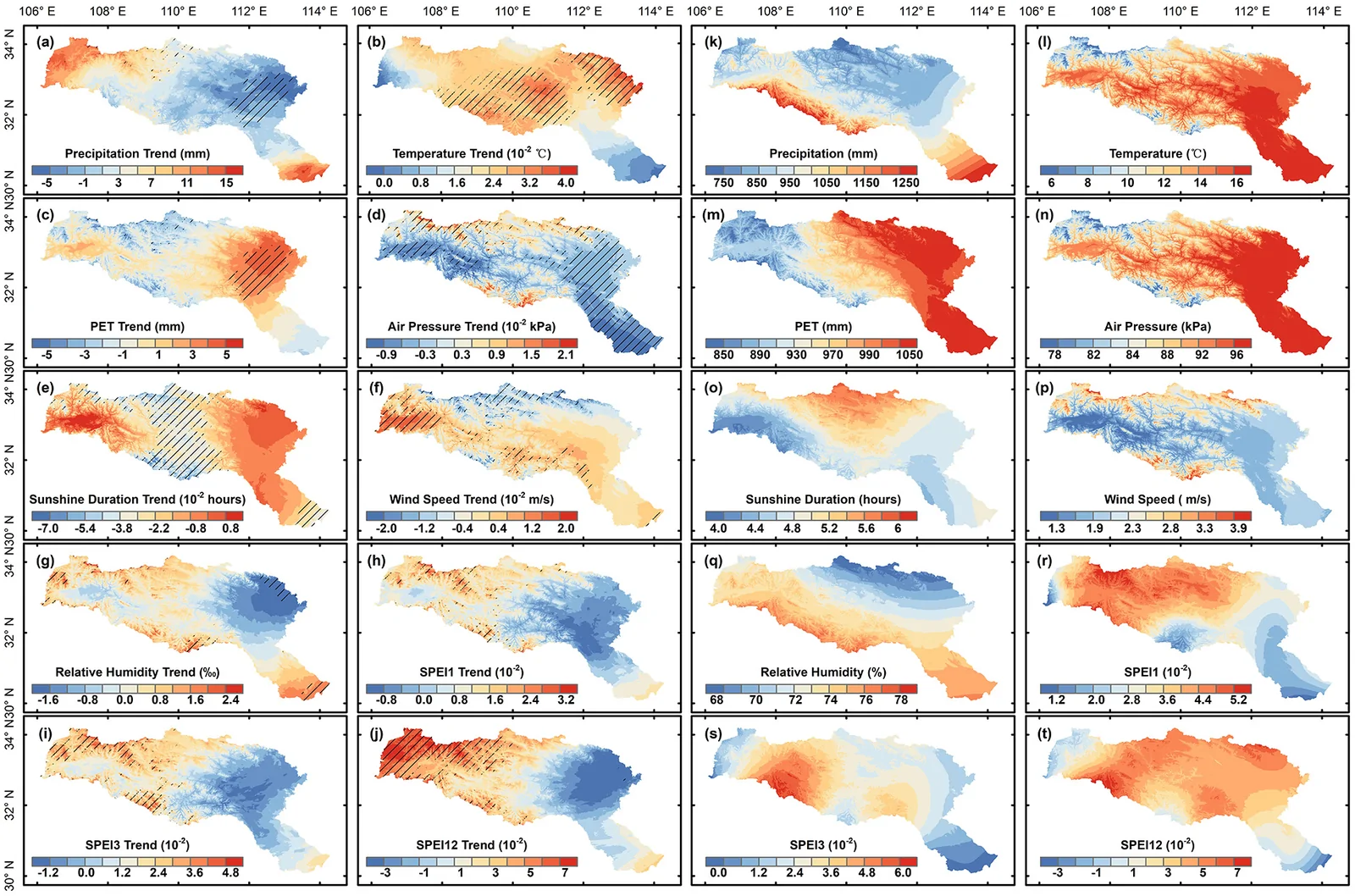
Meteorological factors. **(a–j)** Trends in the climatic indices (slashes indicate significance at the 95% confidence level); **(k–t)** Annual mean values of the climatic indices.

This study used mean values of climate variables and the SPEI over the past 21 years to represent recent climatic conditions in the HJRB, incorporating 14 driving factors: 10 meteorological indices and 4 topographic or anthropogenic variables. For SPEI1 and SPEI3, the annual averages were used as annual summaries of short-term and seasonal drought conditions, rather than as direct representations of individual monthly or seasonal drought events. Beyond the long-term stability of these climatic conditions, trends in climate variables can profoundly influence vegetation, especially under the influences of global warming and the increased frequency of extreme weather events. To characterize the trends for each climate variable, Theil-Sen slopes were utilized. The spatial trends of these climate variables are illustrated in Figure 2.

#### 2.2.3 Topographical data

This study selected two terrain factors, elevation (EV) and slope (S), as the topographic variables. Notably, EV and S are static topographic variables. Therefore, they were used as background environmental variables rather than direct dynamic drivers of GPP trends. In this study, their SHAP contributions were interpreted as the combined effects of terrain-related gradients, including hydrothermal conditions, vegetation-type distribution, soil environment, and human disturbance intensity. The S was calculated based on the digital elevation model (DEM). The DEM data used in this study were obtained from the Shuttle Radar Topography Mission (SRTM) (https://search.earthdata.nasa.gov/search). SRTM provides global coverage of DEM data with a spatial resolution of 90 m. The DEM data from SRTM have been widely utilized in various fields, including terrain analysis, hydrological modeling, geological research, environmental assessment, and remote sensing applications. Consistent with the other factor data, the DEM data were resampled to match the spatial resolution of the GPP data.

#### 2.2.4 Anthropogenic data

##### 2.2.4.1 Land use and land cover (LULC)

LULC data can be used to describe the extent of human activities on natural ecosystems (37, 57, 58). In this study, LU data were sourced from the China Land Cover Dataset (CLCD), a Landsat-derived annual land-cover product for China (https://zenodo.org/record/5816591). This dataset provides comprehensive information on land cover types across China and is widely used for monitoring and researching land cover and land-use dynamics in the country. The data have a spatial resolution of 30 m, and they cover the period from 1990 to 2021 (59). It categorizes land surface cover into nine distinct classes, as detailed in Table 2.

**Table 2:** Land use classification table.

Class	Code
Cropland	1
Forest	2
Shrub	3
Grassland	4
Water	5
Snow/Ice	6
Barren	7
Impervious	8
Wetland	9

**Figure 3:**
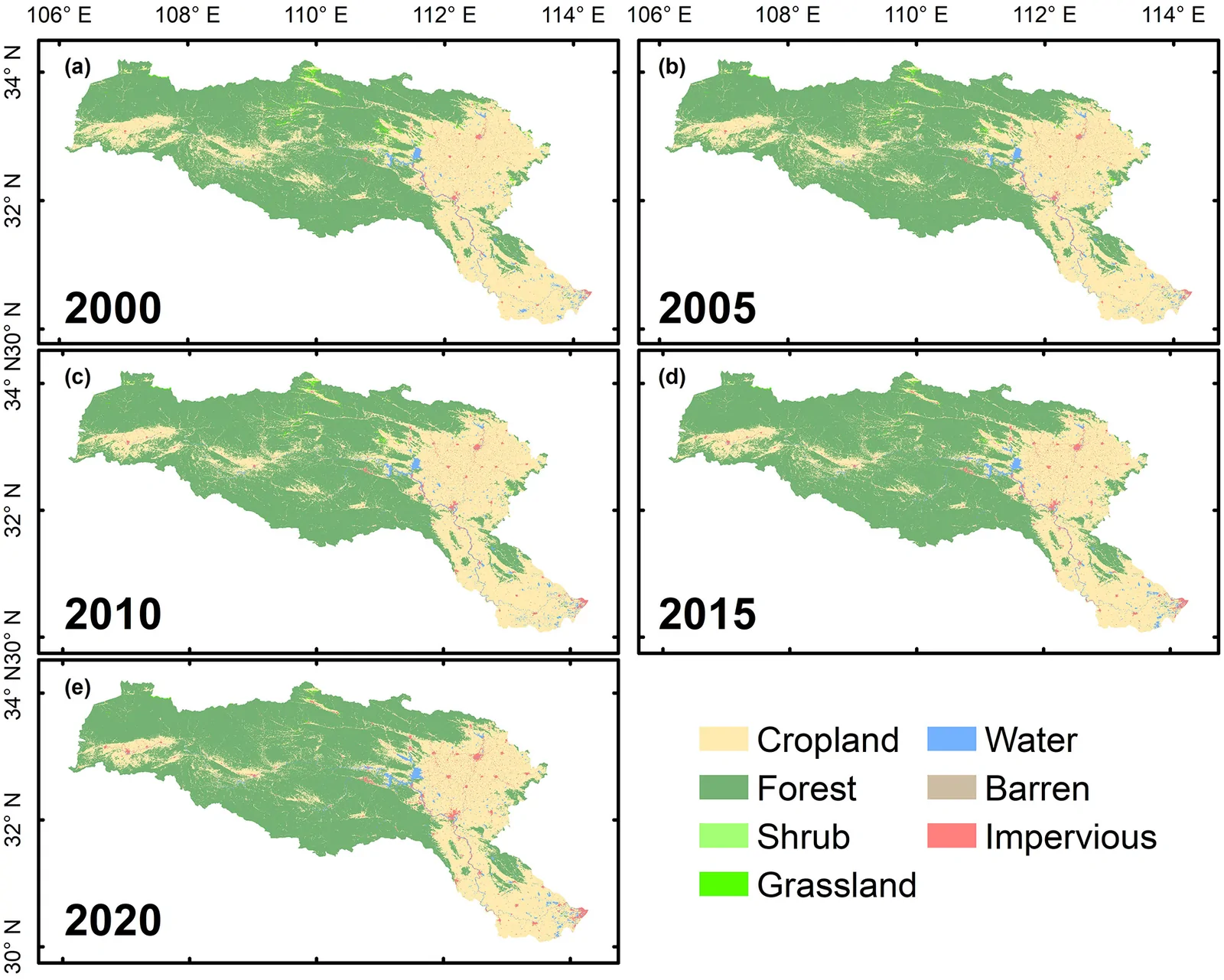
Spatial distribution of land-cover types at five-year intervals: **(a)** 2000; **(b)** 2005; **(c)** 2010; **(d)** 2015; and **(e)** 2020.

The CLCD has been extensively applied to analyze trends and driving factors of change in China, including climate change, anthropogenic activities, ecosystem service assessments, and environmental conservation efforts. To ensure consistency, the LU data were resampled to align with the spatial resolution of the GPP data. The spatial distribution of land-cover types at five-year intervals is shown in Figure 3.

##### 2.2.4.2 Population

Population data were obtained from the LandScan Global population distribution dataset (https://landscan.ornl.gov/), which provides annual ambient population distribution at a native spatial resolution of 30 arc-seconds, approximately 1 km at the equator (60). Population density (PD) was used as a proxy indicator of human activity intensity. To ensure consistency with the GPP and other environmental variables, the annual LandScan data from 2000 to 2020 were projected and resampled onto the common analysis grid using bilinear interpolation because PD is a continuous variable. The mean population density during 2000–2020 was used to represent the average intensity of human activities, and the Theil-Sen slope was calculated to represent the population change trend.

Notably, resampling LandScan data from its native resolution to a finer analysis grid does not generate new population information. This procedure may smooth population-density gradients, especially at urban-rural interfaces and around small construction areas. Therefore, PD was interpreted as an approximate spatial proxy for human activity intensity rather than an exact pixel-scale population exposure variable.

**Figure 4:**
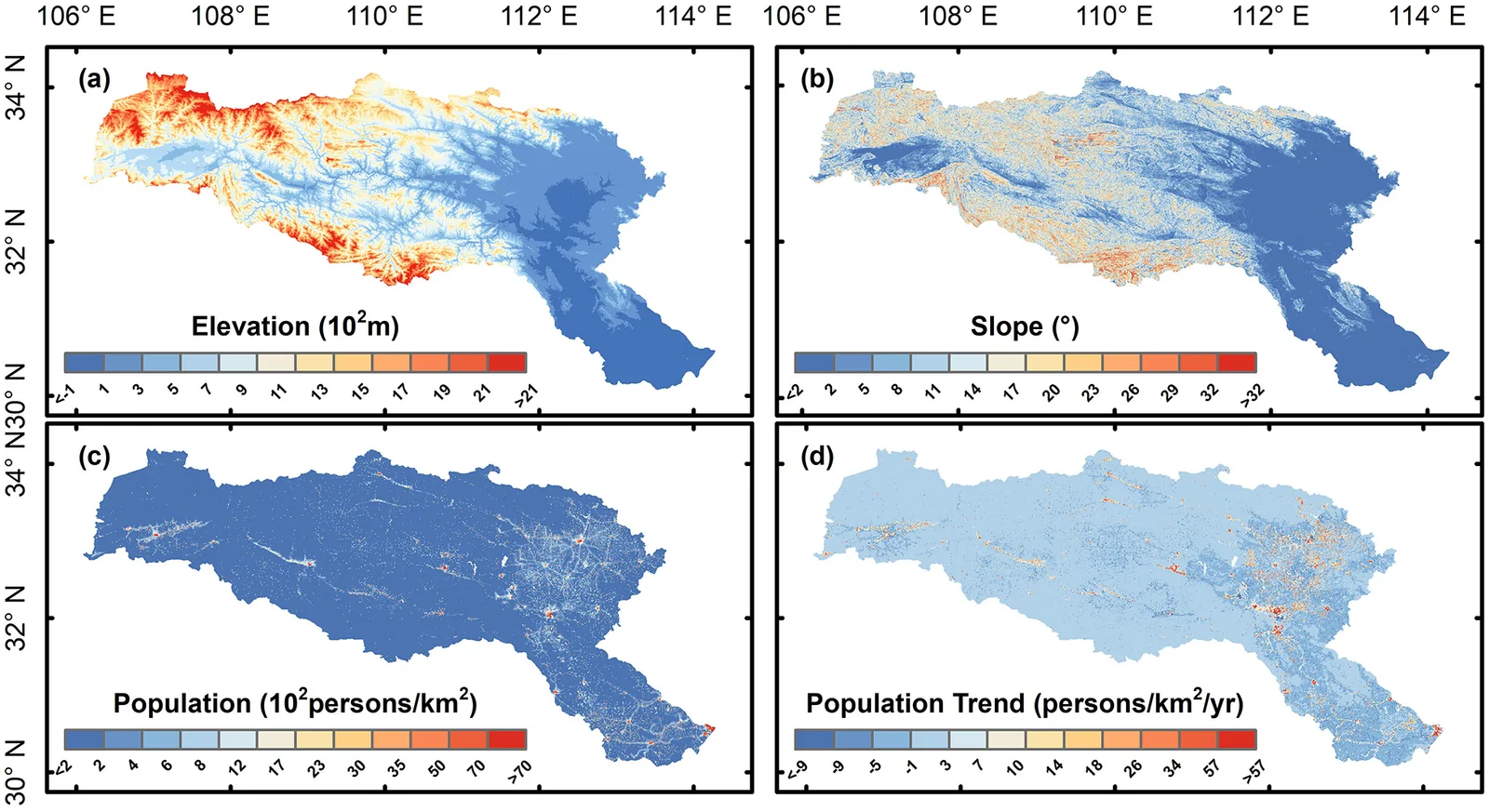
**(a, b)** Topographical factors; **(c)** Annual mean population density; **(d)** Trend in population density.

In this study, LULC and population density were used as spatially explicit proxies for human activity intensity. LULC changes can partly reflect ecological restoration, agricultural adjustment, urban expansion, and reservoir-related land-cover changes, whereas population density represents population pressure and the intensity of human disturbance. However, these variables cannot fully capture the timing, intensity, and management effects of specific ecological restoration projects, water-diversion engineering, or fine-scale urban expansion. Therefore, the anthropogenic variables in this study should be interpreted as broad proxies of human activity rather than complete measurements of all human interventions. The spatial distributions of elevation, slope, mean population density, and the population-density trend are shown in Figure 4.

### 2.3 Method

**Figure 5:**
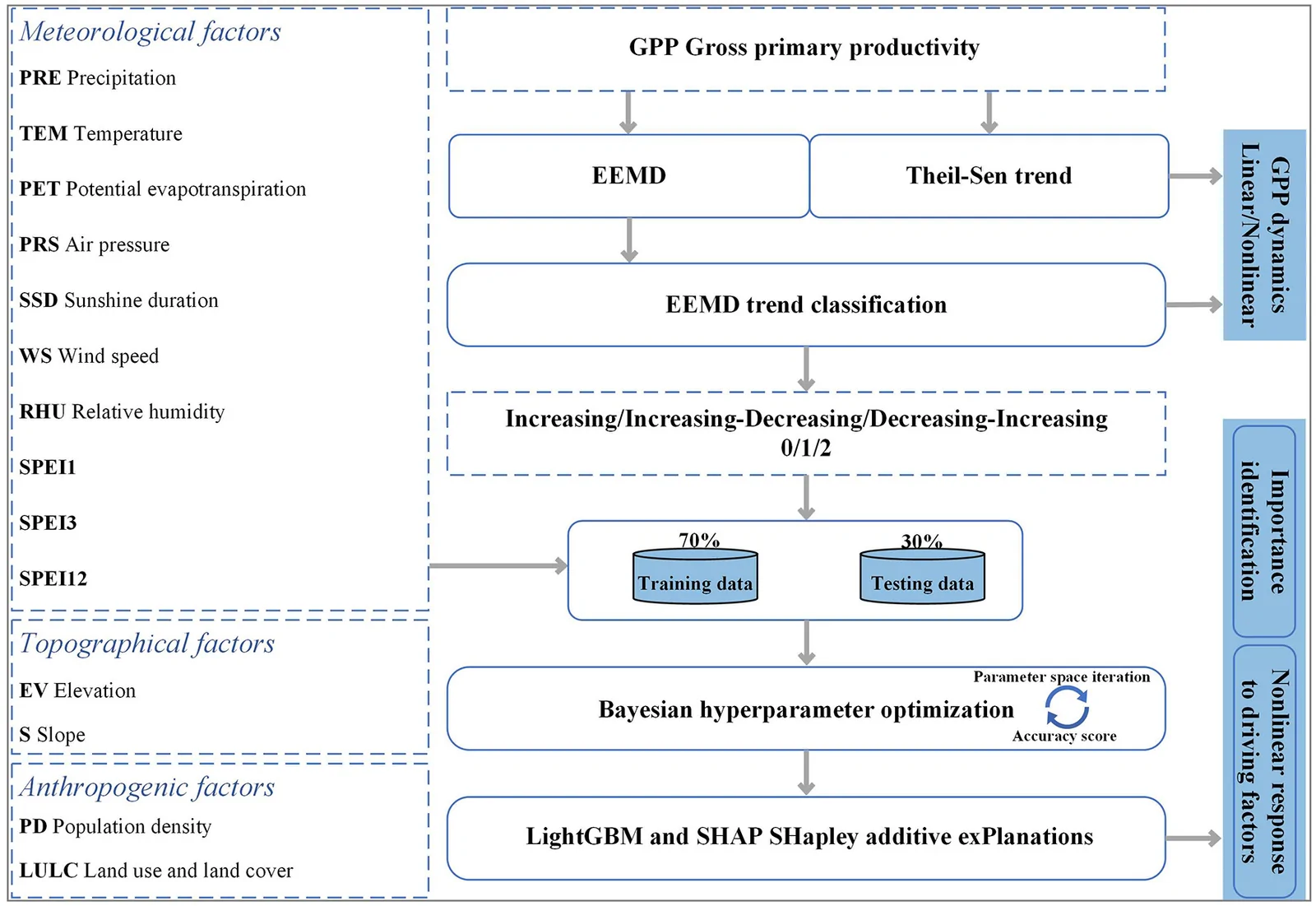
The framework of methodology and analytical procedure in this study.

The framework of the methodology and analytical procedures in this study is shown in Figure 5. The procedures include constructing the training and testing datasets, classifying GPP trends, optimizing hyperparameters, interpreting the model, and analyzing and discussing the results.

#### 2.3.1 Theil-Sen trend analysis

The Theil–Sen trend analysis method is an emerging non-parametric statistical method that estimates trends in an entire time series by calculating the median of the slopes between all pairs of points (61). The advantage of this method is that it does not require the samples to follow a specific distribution and is not influenced by outliers. Compared to the ordinary least squares (OLS) based greenness rate-of-change method, the Theil-Sen trend analysis method exhibits higher robustness, effectively eliminating the influence of outliers and extreme values while requiring no assumptions about the data (42, 62). The formula for the Theil–Sen method is given in Equation 1:


$$\begin{array}{l} \beta =Median\left(\frac{x_{j}-x_{i}}{j-i}\right),\forall j>i \end{array}$$ (1)

Where β represents the vegetation change trend; i and j are time indices; and GPP corresponds to the GPP values at times i and j. If β > 0, it indicates an increasing trend; if β < 0, it indicates a decreasing trend. Additionally, the Mann–Kendall test was used to assess the significance of time-series trends in this study (63). When the absolute value exceeds 1.96, it indicates that the trend has passed the 95% significance test.

#### 2.3.2 EEMD

EEMD, proposed by Wu and Huang, is a time-series data decomposition method that effectively removes nonlinear and non-stationary components, enabling the extraction of underlying temporal trends (22). To address mode mixing in EMD (23) and enhance the robustness of the decomposition results, EEMD adds white noise to the original data and determines the ensemble mean of the decompositions. The specific steps of the EEMD calculation are as follows:

(1) Gaussian white noise was iteratively added to the input data *x*(*t*), as shown in Equation 2:


$$\begin{array}{l} x_{1}\left(t\right)=w_{1}\left(t\right)+x\left(t\right) \end{array}$$ (2)

(2) The local maxima and minima were connected using cubic splines to form the upper and lower envelopes. The mean envelope *m*_1_(*t*) and the first proto-IMF *h*_1_(*t*) were calculated using Equations 3 and 4, respectively:


$$\begin{array}{l} m_{1}\left(t\right)=\left[e_{u}\left(t\right)+e_{1}\left(t\right)\right]/2 \end{array}$$ (3)

$$\begin{array}{l} h_{1}\left(t\right)=x_{1}\left(t\right)-m_{1}\left(t\right) \end{array}$$ (4)

(3) The function is evaluated to determine if it satisfies the condition of being sufficiently close to zero at every point. If the condition is met, the computation is stopped. However, if the condition is not satisfied, it is treated as new time-series data, and step 2 is repeated iteratively until the average value of the k-th envelope meets the stopping criterion. The second sifting result is calculated using Equation 5, and, after *k* iterations, the first intrinsic mode function (IMF), *C*_1_(*t*), is obtained using Equation 6:


$$\begin{array}{l} h_{2}\left(t\right)=h_{1}\left(t\right)-m_{2}\left(t\right) \end{array}$$ (5)

$$\begin{array}{l} C_{1}\left(t\right)=h_{k}\left(t\right)=h_{k-1}\left(t\right)-m_{k}\left(t\right) \end{array}$$ (6)

(4) After the second IMF is extracted, the second residual, *R*_2_(*t*), is calculated by subtracting *C*_2_(*t*) from *R*_1_(*t*), as shown in Equation 7. This procedure is repeated iteratively, and the /(*n*)/-th residual, *R*_*n*_(*t*), is calculated using Equation 8, until it contains no further oscillatory components:


$$\begin{array}{l} R_{2}\left(t\right)=R_{1}\left(t\right)-C_{2}\left(t\right) \end{array}$$ (7)

$$\begin{array}{l} R_{n}\left(t\right)=R_{n-1}\left(t\right)-C_{n}\left(t\right) \end{array}$$ (8)

At this stage, *x*_1_(*t*) was decomposed into n intrinsic mode functions (IMFs) with decreasing frequencies and a residual trend component that either contained a single extremum or was entirely monotonic, as expressed in Equation 9:


$$\begin{array}{l} x_{1}\left(t\right)=\overset{n}{\underset{j=1}{\sum }}C_{j}\left(t\right)+R_{n}\left(t\right) \end{array}$$ (9)

(5) The above steps are repeated i times by adding different Gaussian white noise sequences to *x*_1_(*t*), and the corresponding means of the IMFs and residual trend are taken as the final results.

(6) After considering the computational time and robustness of the results, this study set the number of added Gaussian white noise instances to 100. Experimental results have shown that increasing the number of noise instances yields only marginal differences and does not offset the linear increase in computational time (18). The amplitude of the white noise was set to 0.1 times the standard deviation of the original data (18, 20, 22).

After EEMD decomposition, the residual component R(t) was extracted as the nonlinear long-term trend of annual GPP for each pixel. The trend type was determined according to the direction of change in the residual component and the presence of a turning point. First, the overall change was calculated as Δ*R* = *R*(*t*_*end*_) − *R*(*t*_*start*_). To avoid classifying weak fluctuations as trend transitions, a minimum change magnitude threshold was set as 2% of the residual range, i.e., *δ* = 0.02 × [max(*R*)−min(*R*)]. Changes smaller than this threshold were regarded as weak fluctuations.

For each pixel, a potential turning point was identified from the residual series as the year at which the difference between the mean residual values before and after the candidate year reached its maximum. Candidate turning points were searched only within the internal years of the time series to avoid unstable endpoints. The slopes before and after the turning point were then compared to determine whether the residual trajectory showed a monotonic trend or a reversal. Based on this rule, four EEMD trend types were classified: (1) increase, where the residual component showed an overall positive change without a reversal; (2) decrease, where the residual component showed an overall negative change without a reversal; (3) increase decrease, where the residual first increased and then decreased after the turning point; and (4) decrease increase, where the residual first decreased and then increased after the turning point.

The EEMD trend type classification was rule-based and focused on the shape of the nonlinear residual trajectory. Statistical significance was not used as a direct criterion for assigning EEMD trend types. Instead, the Mann–Kendall significance test was applied to the Theil–Sen linear trend analysis as an independent reference for the overall monotonic trend. Therefore, the EEMD classification should be interpreted as a nonlinear trajectory classification rather than a statistical significance test of trend change.

#### 2.3.3 LightGBM

LightGBM, a machine learning approach introduced by Ke et al., is a gradient boosting decision tree (GBDT) method. It addresses the challenges of large data volume and high feature dimensionality in GBDT by incorporating two innovative techniques: gradient-based one-side sampling (GOSS) and exclusive feature bundling (EFB). The GOSS algorithm effectively reduces the number of training samples, thereby accelerating model training. Furthermore, because influential samples contribute significantly to the model's performance, preserving all the influential samples enhances the predictive accuracy. By leveraging this sampling approach, the GOSS algorithm achieves computational efficiency without compromising the model's effectiveness.

In the GOSS method, the instances of a × 100% with larger gradients are initially allocated to subset A. Subsequently, a new subset B is created by randomly sampling instances from the remaining set *A*^*C*^, which includes the instances from subset (1–a) × 100% and consists of instances with smaller gradients. The sampling is conducted with a specified size *b* × |*A*^*C*^|. Finally, the variance gain is utilized to facilitate the partitioning of data instances, as demonstrated by the subsequent Equation 10:


$$\begin{array}{l} {\tilde{V}}_{j}\left(d\right)\\ =\frac{1}{n}\left(\frac{\sum_{x_{i}\in A_{l}}g_{i}+\frac{1-a}{b}\sum_{x_{i}\in B_{l}}g_{i}}{n_{l}^{i}\left(d\right)}+\frac{\sum_{x_{i}\in A_{r}}g_{i}+\frac{1-a}{b}\sum_{x_{i}\in B_{r}}g_{i}}{n_{r}^{i}\left(d\right)}\right) \end{array}$$ (10)

Where $n_{l}^{i}\left(d\right)$ and represent the number of instances in the left and right nodes, respectively; $\frac{1-a}{b}$ denotes the coefficient for gradient normalization and corresponds to subsets of subset. Similarly, present subsets of subset resub sets of subsets *B*.

The EFB algorithm reduces feature space and complexity through feature selection, feature encoding, and feature bundling. This reduction not only decreases the training time and memory consumption of the model but also helps prevent overfitting and improves its generalization ability. For a more detailed computation process of the LightGBM model, refer to Ke et al.

Before model interpretation, both Pearson and Spearman correlation analyses were conducted for the climatic and hydrometeorological variables to diagnose pairwise collinearity. Furthermore, variance inflation factor (VIF) tests were used to quantify multicollinearity among these variables. Considering that SPEI is derived from precipitation and potential evapotranspiration and, therefore, may be mathematically correlated with PRE and PET, the SHAP results were not interpreted as independent causal contributions of individual climatic variables. To further evaluate the robustness of feature importance rankings, collinearity sensitivity tests were conducted by retraining the LightGBM model under three feature settings: the full feature set, a feature set excluding SPEI variables, and a feature set excluding PRE and PET variables. The Pearson/Spearman correlation matrix, VIF results, and sensitivity tests are provided in Supplementary Figure S1, Supplementary Tables S1, S2, respectively.

The determination of hyperparameters has a significant impact on the predictive performance of the model (64). In this study, a Bayesian optimization method for hyperparameter tuning was employed to efficiently explore the hyperparameter space and automatically adjusted the hyperparameters to enhance the model's performance (65). Compared with traditional grid search or random search, Bayesian optimization can find better hyperparameter combinations in fewer iterations, thereby saving time and computational resources (66). In this study, a 10-fold cross-validation was conducted using the Bayesian search method for parameter optimization, with accuracy selected as the objective function for evaluation. The initial hyperparameter space and optimized parameter results are summarized in Table 3. After obtaining the optimized parameters, 70% of the pixels from the effective regions were used as the training dataset, while the remaining 30% were reserved as the test dataset for training the LightGBM model. The original LU code was retained only for zonal statistics and interpretation.

**Table 3:** Hyperparameter space constructed.

Hyperparameter	Parameter space	Best parameter
learning_rate	Real (0.1, 1.0, 'log uniform')	0.1391
n_estimators	Integer (100, 5000)	2,631
num_leaves	Integer (15, 512)	69
max_depth	Integer (1, 256)	56
subsample	Real (0.01, 1.0, 'uniform')	0.8973
subsample_freq	Integer (1, 10)	3
colsample_bytree	Real (0.01, 1.0, 'uniform')	0.7740
reg_alpha	Real (1e9, 100.0, 'uniform')	45.0838
reg_lambda	Real (1e9, 100.0, 'uniform')	80.8911

Because pixel-wise samples may exhibit spatial autocorrelation, random partitioning may lead to spatial overlap between training and testing samples, thereby overestimating model performance. To further evaluate model generalizability, we conducted spatial block cross-validation. The study area was divided into 0.1° × 0.1° spatial blocks, and entire blocks rather than individual pixels were assigned to different folds. Five-fold spatial block cross-validation was then performed for the three major EEMD trend types used in the LightGBM-SHAP analysis. Random five-fold cross-validation was also conducted for comparison. Because land-cover type is categorical, LULC was transformed into one-hot encoded variables before model training to avoid imposing artificial ordinality among land-cover classes. The original LU code was retained only for zonal statistics and interpretation. Land-cover transition codes were constructed by combining the initial and final land-cover classes; for example, code 18 represents conversion from cropland to impervious surface.

#### 2.3.4 SHapley additive explanations (SHAP)

SHAP is an additive explanation model developed by Lundberg and Lee, inspired by cooperative game theory. In this model, all features are considered “contributors.” For each prediction sample, the model generates a prediction value, and the SHAP value represents the numerical allocation of each feature in that sample. SHAP is based on an additive feature attribution method, where a linear function g of binary variables is constructed to estimate the target function f, as expressed in Equation 11:


$$\begin{array}{l} f\left(x\right)\approx g\left(z^{*}\right)=\varphi_{0}+\overset{M}{\underset{i=1}{\sum }}\varphi_{i}z_{i}^{*} \end{array}$$ (11)

Here, *z* = {0, 1}^*M*^ is a binary vector indicating the presence or absence of each input feature, and *M* is the total number of input features. The SHAP value of feature *i*, denoted by *φ*_*i*_, is calculated using Equation 12:


$$\begin{array}{l} \phi_{i}=\underset{S\subseteq N\backslash \left\{i\right\}}{\sum }\frac{|S|!\left(M-|S|-1\right)!}{M!}\left[f_{x}\left(S\cup \left\{i\right\}\right)-f_{x}\left(S\right)\right] \end{array}$$ (12)

Where *N* represents the set of all input features; *S* represents the subset of different feature combinations without feature; and *i* represents the predictive model with feature-combination subset conditioned on input instance. For TreeSHAP, is defined as the additive value of *f* for the input feature subset. Traditional feature importance measures indicate only which features are important without reflecting how they affect the prediction. Conversely, SHAP values not only reflect the influence of each feature on each sample but also indicate whether the feature has a positive or a negative impact.

Once the feature importance is calculated using SHAP values, visualizations can be generated. The average absolute SHAP value for all outputs represents their global impact. Additionally, SHAP dependency plots can show how features influence predictions, revealing the relationship between GPP variations and their influencing factors. Since SHAP generates a prediction value for each sample by default, the output dependency plot is presented as a scatter plot. To explore differences among trends in GPP and the nonlinear relationship between influencing factors and GPP, this study used a non-parametric regression technique, locally weighted scatterplot smoothing (LOWESS) (67), to smooth the scatter plot into curves. In this study, the EEMD-derived nonlinear trend type of GPP was used as the response variable in the LightGBM model. Therefore, SHAP values should be interpreted as the contribution of each climatic, topographic, or anthropogenic factor to the model output for a given EEMD trend type, rather than as a direct change in GPP magnitude. Specifically, positive SHAP values indicate that a factor contributes positively to the model prediction of a certain nonlinear trend type, whereas negative SHAP values indicate a negative contribution to the model prediction of that trend type. This analytical design links the temporally nonlinear information extracted by EEMD with the spatial environmental gradients represented by climatic and human activity variables, thereby allowing the response pathways of different GPP trend types to be interpreted separately.

#### 2.3.5 Zonal statistics for sub-basin and land cover heterogeneity

To further examine the spatial heterogeneity of nonlinear GPP trends, zonal statistics were computed by overlaying the EEMD-derived GPP trend-type map with the upper- and lower-reach boundaries of the HJRB and the land-cover map. For each sub-basin and land cover class, the pixel numbers and proportions of the four EEMD trend types, including continuous increase, continuous decrease, increase to decrease, and decrease to increase, were calculated. This analysis was used to identify whether the dominant nonlinear GPP trends differed among basin subregions and major land cover types. The corresponding zonal statistics are summarized in Table 4.

**Table 4:** Zonal statistics of nonlinear GPP trend types in the upper and lower reaches of the HJRB.

Region	Decrease	Decrease to increase	Increase	Increase to decrease
Upper reach	0.56%	4.51%	71.69%	23.25%
Lower reach	0.87%	4.25%	58.74%	36.14%

To clarify the methodological contribution of the proposed framework, we compared it with commonly used approaches in vegetation trend attribution (Supplementary Table S3).

## 3 Results

### 3.1 Spatiotemporal characteristics of GPP dynamics

**Figure 6:**
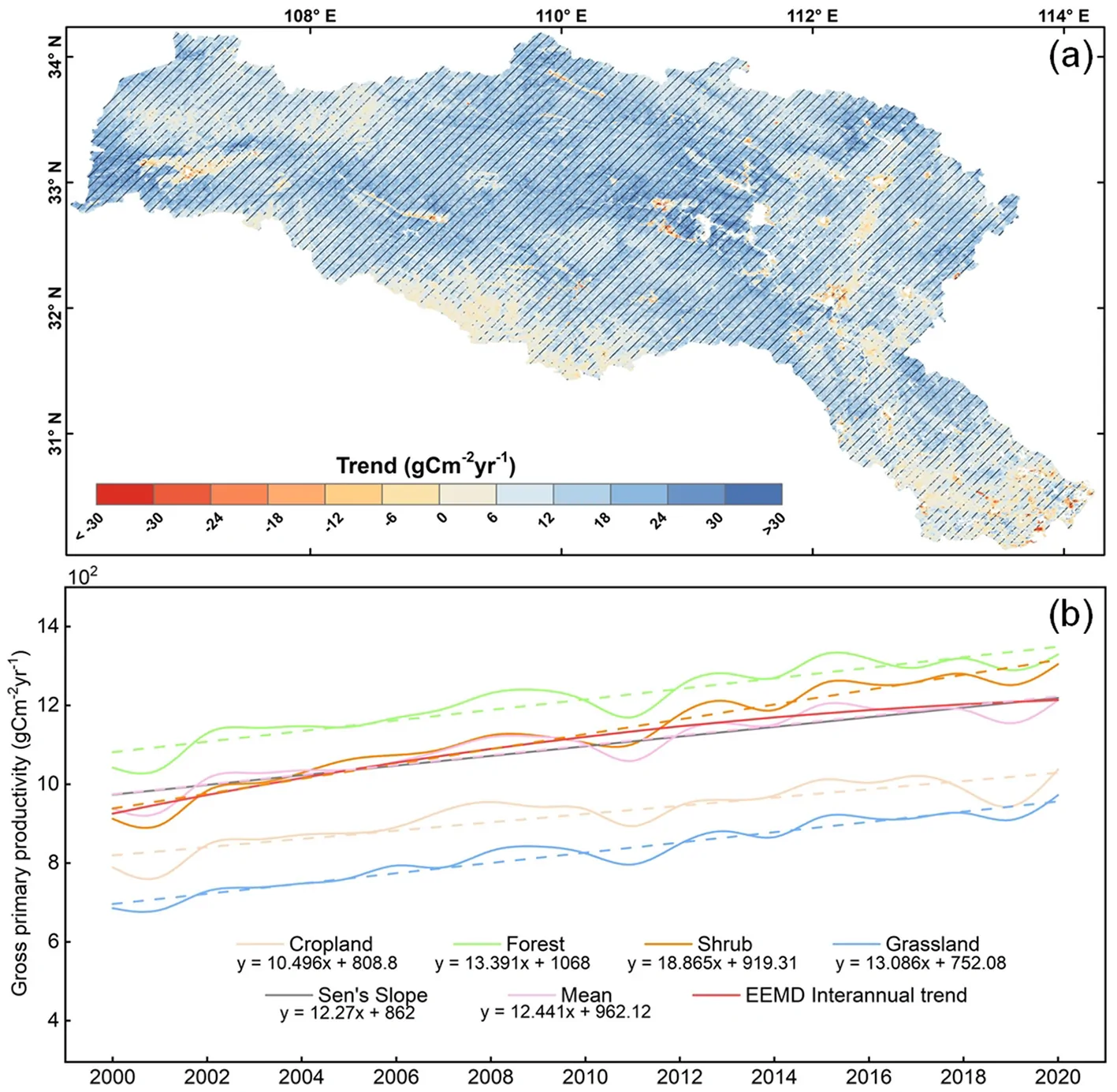
**(a)** Spatial distribution of the Theil–Sen trend of GPP in the HJRB (slash means passed the 95% significance test); **(b)** Interannual variation in GPP for different land uses in the HJRB.

Between 2000 and 2020, the GPP of the HJRB fluctuated from 897.34 to 1,223.21 g C m^−2^ yr^−1^, with an overall mean of 1,095.61 g C m^−2^ yr^−1^. The minimum GPP occurred in 2001, whereas the maximum occurred in 2015. The Theil-Sen analysis yielded an increasing rate of 12.27 g C m^−2^ yr^−1^ (*P* < 0.01), indicating a significant overall increase in GPP. The spatial analysis of the Theil-Sen trend showed that 97.22% of the HJRB region exhibited a growth trend (Figure 6).

Among the various land cover types within the HJRB, the mean GPP of forests from 2000 to 2020 was 1,215.27 g C m^−2^ yr^−1^, which was higher than that of other land cover types. The GPP fluctuations of all land cover types showed a similar trend (Figure 6). Based on linear fitting, the Theil-Sen trend, and EEMD analysis, the HJRB exhibited a significant increasing trend between 2000 and 2020. However, the linear and Theil-Sen trends can capture only a stable trend in GPP changes during specific time intervals, whereas EEMD can highlight the detailed evolutionary process of GPP over time.

**Figure 7:**
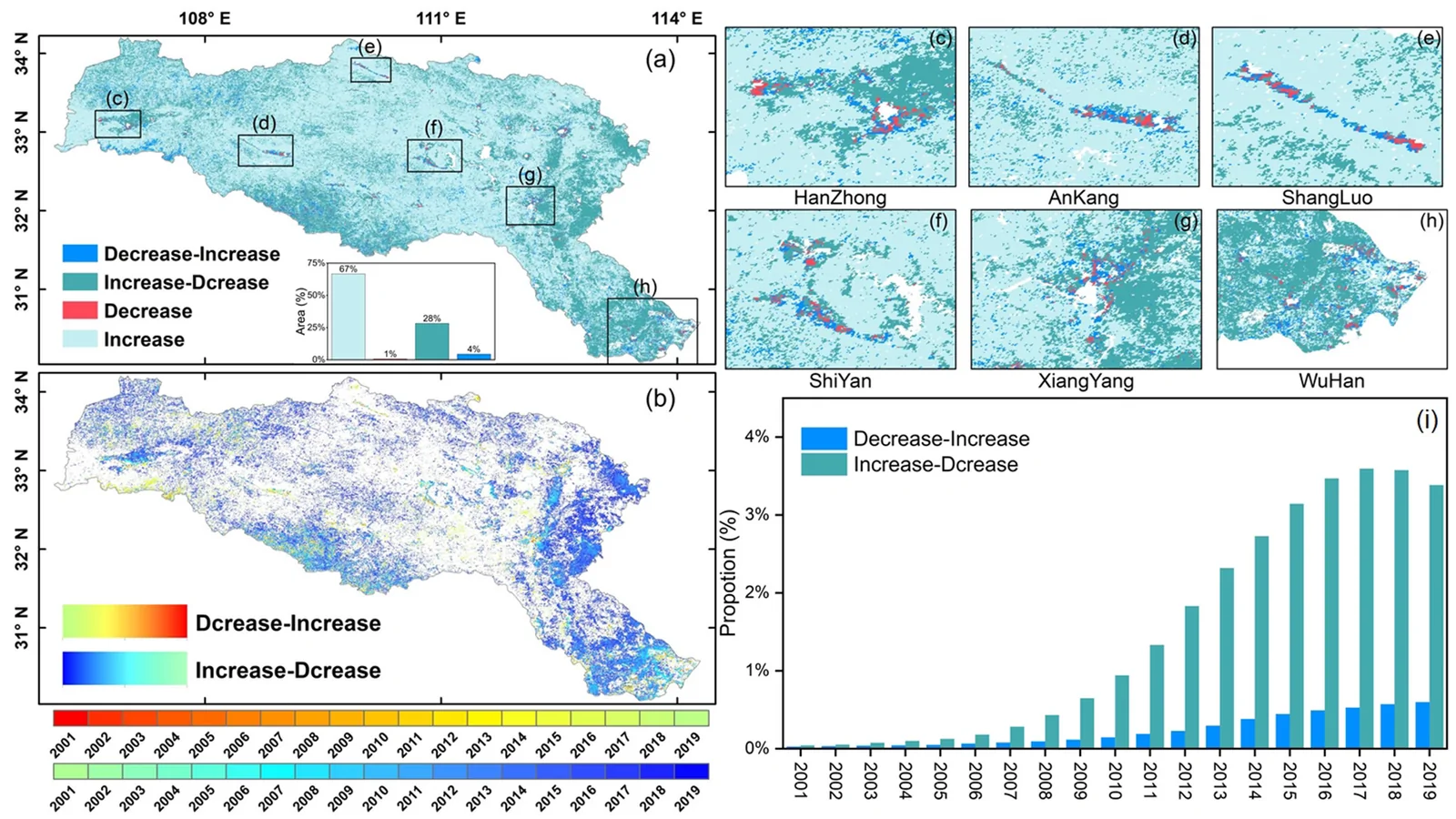
EEMD analysis results. **(a)** Spatial distribution and area proportions of the EEMD-derived GPP trend types in the HJRB; **(b)** spatial distribution of breakpoint years for the decrease-to-increase and increase-to-decrease trend types; **(c–h)** enlarged views of the boxed regions in panel **(a)**, corresponding to Hanzhong, Ankang, Shangluo, Shiyan, Xiangyang, and Wuhan, respectively; and **(i)** annual area proportions of pixels with identified breakpoints for the decrease-to-increase and increase-to-decrease trend types from 2001 to 2019. A breakpoint denotes the turning year identified from the EEMD residual component.

To further link the land cover differences shown in Figure 6 with nonlinear GPP trends, zonal statistics were conducted for major land cover types. Forests showed the highest proportion of continuous increase among the dominant land cover classes, accounting for 71.76%, whereas cropland had a lower proportion of continuous increase (58.63%) and a higher proportion of increase-to-decrease trends (36.29%). Grassland was also dominated by continuous increase (87.75%), although its spatial extent was relatively limited. Conversely, barren and impervious surfaces showed a much higher proportion of continuous decrease (5.65%) than the basin average, indicating that negative GPP changes were more concentrated in areas affected by intensive human disturbance. Based on the residual component extracted by EEMD and the above trend-type classification rules, the GPP trajectories were divided into four nonlinear trend types: increase, decrease, increase-to-decrease, and decrease-to-increase. The EEMD residual analysis indicated an overall upward trend in the GPP of the HJRB from 2000 to 2020, with a deceleration in the growth rate over time (Figure 7b). Pixel-wise calculations can reflect the spatial variations in GPP trends. The pixel-wise EEMD analysis revealed that 66.67% of the region exhibited a continuous upward trend, primarily distributed around the Danjiangkou Reservoir in the middle reach of the HJRB and in the upstream and downstream mountainous areas. The spatial correspondence between the continuous increase in GP and areas around the Danjiangkou Reservoir and in mountainous ecological conservation zones suggests that regional ecological protection and restoration activities may have contributed to sustained improvements in vegetation productivity. However, their effects cannot be fully separated from climate variability using the current proxy variables. Additionally, only 0.68% of the region exhibited a decreasing trend, primarily around areas designated for construction (Figures 7e–h). Although this continuous decreasing type occupied only a very small proportion of the basin, its spatial concentration around construction and urbanized areas suggests that it may represent localized but ecologically important negative effects of human activities, especially urban expansion and the expansion of impervious surfaces. Furthermore, 28.25% of the region had an increasing trend followed by a decreasing trend, and these areas were mainly distributed in the southern part of the upstream region and the eastern part of the downstream region. Moreover, 4.41% of the region had a decreasing trend followed by an increasing trend, and these areas were scattered throughout the HJRB (Figure 7a). Spatially, the downstream region exhibited a broader distribution of trends transitioning from increasing to decreasing, with breakpoints occurring later, whereas the upstream region contained both trend types, with earlier breakpoints (Figure 7b). The transition from increasing to decreasing trends showed a sharp rise starting in 2000, followed by a gradual decline post 2017; meanwhile, the transition from decreasing to increasing trends exhibited a slow upward trend beginning in 2000 (Figure 7i).

Zonal statistics further revealed clear spatial heterogeneity between the upper and lower reaches of the HJRB. In the upper reach, the continuous increase type accounted for 71.69%, while the increase-to-decrease type accounted for 23.25%. Conversely, the lower reach showed a lower proportion of continuous increase (58.74%) and a much higher proportion of increase-to-decrease trend (36.14%). This suggests that the weakening or reversal of GPP growth was more pronounced in the lower reach.

### 3.2 Importance identification of driving factors

In this study, the LightGBM model was employed to investigate the relationship between the HJRB GPP and its driving factors. Considering that the continuous decreasing trend in GPP accounted for only 0.68% of the entire HJRB, its sample size was too small to support stable multiclass learning and SHAP interpretation. Therefore, to avoid severe class imbalance and unreliable feature attribution, the LightGBM SHAP analysis focused on three major EEMD trend types: increasing, increase-to-decrease, and decrease-to-increase. However, excluding the continuous decrease type from the machine-learning attribution does not imply that this type is ecologically unimportant. Because this type was primarily observed in construction and urbanized areas, it was further interpreted qualitatively in the Discussion as a potential signal of localized negative human impacts. The performance of the final LightGBM model is presented in Table 5. The testing AUC of 0.7997 and precision of 0.7541 indicate that the model had a moderate ability to distinguish among the major EEMD trend types when making positive predictions.

**Table 5:** Performance of the LightGBM model for classifying the three major EEMD trend types.

Dataset	Precision	AUC	F1 score	Recall
Training	0.7884	0.8723	0.5637	0.5207
Testing	0.7541	0.7997	0.5025	0.4732

Precision and AUC indicate the reliability and discriminative power, whereas recall and F1 score reflect the ability to correctly identify all samples of each trend type. The relatively low recall and F1 score indicate limited identification ability for some nonlinear trend types.

**Figure 8:**
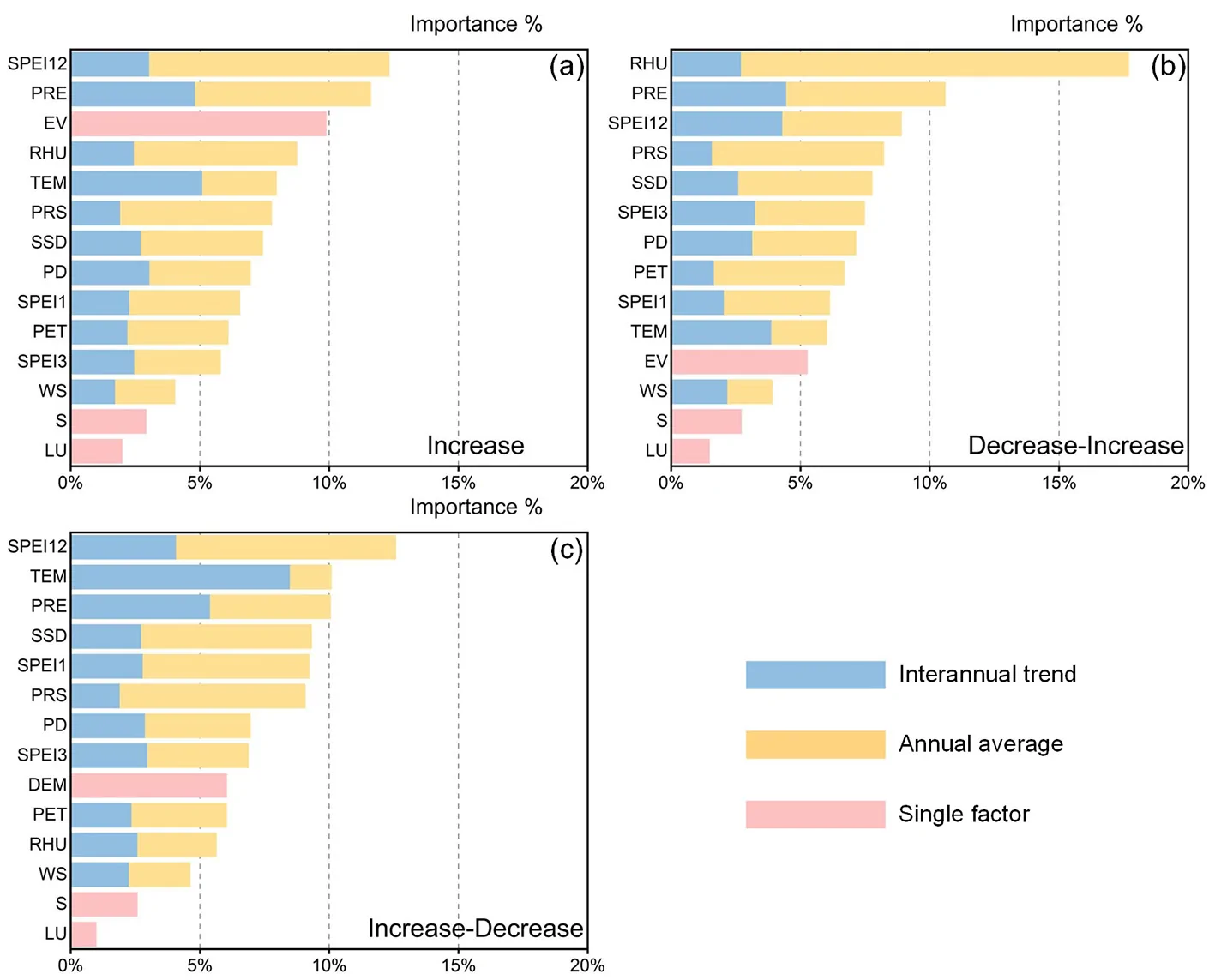
SHAP-based importance of individual parameters for each EEMD-derived trend type: **(a)** continuous increase; **(b)** decrease-to-increase; and **(c)** increase-to-decrease. Blue, yellow, and pink bars represent interannual trends, annual averages, and single factors, respectively.

To account for potential spatial autocorrelation, spatial block cross-validation was further conducted. Compared with random five-fold cross-validation, spatial block cross-validation produced slightly lower but comparable performance, with macro-AUC decreasing from 0.7353 to 0.7204, macro-F1 decreasing from 0.4925 to 0.4844, and balanced accuracy decreasing from 0.5210 to 0.5054. This indicates that random partitioning may slightly overestimate model performance because of spatial autocorrelation. Therefore, the LightGBM-SHAP results were interpreted mainly as evidence of factor importance and nonlinear associations rather than as highly accurate pixel-level classification. However, the relatively low F1 score (0.5025) and recall (0.4732) suggest that the model had limited sensitivity in identifying all samples of some nonlinear trend types, especially minority or transitional classes. Therefore, the LightGBM results were used mainly to support the interpretation of factor importance and nonlinear response patterns, rather than to claim highly accurate classification of all GPP trend types. Subsequently, the SHAP values for all features in the model were computed to represent the importance of each feature and quantify its contribution. Figure 8 and Table 6 illustrate the relative importance of each factor in the model for each EEMD trend.

**Table 6:** Relative importance of driving factors to different EEMD trends.

Factor types	ID	Relative importance (%)			Average importance (%)	Total importance (%)
		Increasing	Increasing to decreasing	Decreasing to increasing		
Meteorological	SPEI12	12.32	12.57	8.90	11.27	81.71
	PRE	11.61	10.05	10.60	10.75	
	RHU	8.74	5.64	17.70%	10.69	
	PRS	7.77	9.07	8.22	8.35	
	SSD	7.42	9.32	7.77	8.17	
	TEM	7.96	10.08	6.01	8.02	
	SPEI1	6.54	9.23	6.13	7.30	
	SPEI3	5.79	6.87	7.48	6.71	
	PET	6.09	6.03	6.69	6.27	
	WS	4.03	4.62	3.91	4.19	
Anthropogenic	PD	6.95	6.95	7.15	7.02	8.50
	LU	1.99	0.99	1.47	1.48	
Topographical	DEM	9.88	6.03	5.26	7.06	9.79
	S	2.91	2.57	2.71	2.73	

Among the 14 driving factors analyzed, SPEI12 and PRE emerged as highly significant in influencing vegetation change trends. SPEI12 ranked first, third, and first for the increasing, decrease-to-increase, and increase-to-decrease trends, respectively, with relative importance values of 12.32%, 8.90%, and 12.57%. Similarly, PRE was a critical factor, ranking second for both the increase and decrease to increase trends and third for the increase-to-decrease trend, with relative importance values consistently exceeding 10% (11.61%, 10.60%, and 10.05%). Conversely, TEM ranked fifth, tenth, and second in the three trends, respectively, with its importance exceeding 10% (10.08%) only in the increasing to decreasing trend, mainly due to the interannual variation (contributing 72.74%), which differed from other climate factors (Table 6, Figure 8). Additionally, drought (SPEI1, SPEI3, and SPEI12) had a substantial impact on GPP trends, with relative importance values of 24.65%, 22.51%, and 28.67%, respectively. However, the influence of drought on the HJRB GPP trend mainly manifested in long-term drought (SPEI12) (Table 6, Figure 8). In the decreasing to increasing trend, RHU ranked first with a relative importance of 17.70%, which was significantly higher than that of other factors, but it ranked fourth and 11th in the increasing and increasing to decreasing trends, respectively (Table 6, Figure 8). This indicates that atmospheric moisture conditions, rather than precipitation amount alone, were particularly important for distinguishing areas where GPP shifted from decline to recovery. PD, SSD, PRS, and PET had moderate importance levels across the three trends. PD ranked eighth, seventh, and seventh in the three trends, with relative importance values of 6.95%, 7.15%, and 6.95%, respectively. SSD ranked seventh, fifth, and fourth in the three trends, with relative importance values of 7.42%, 7.77%, and 9.32%, respectively. PRS ranked sixth, fourth, and sixth in the three trends, with relative importance values of 7.77%, 8.22%, and 9.07%, respectively. PET ranked 10th, eighth, and 10th in the three trends, with relative importance values of 6.09%, 6.69%, and 6.03%, respectively (Table 6, Figure 8). Notably, DEM ranked third in the continuously increasing trend, whereas its contribution to the other two nonlinear trend types was relatively limited. Since DEM is a static topographic variable, this result should not be interpreted as a direct causal effect of elevation on GPP dynamics. Instead, DEM likely reflects an integrated environmental gradient associated with climate conditions, vegetation types, soil properties, and the intensity of human disturbance. Similarly, LU, S, and WS did not exhibit significant importance across the three trends. Therefore, the SHAP values in this study should be interpreted as the contribution of each driving factor to the model output for the three major EEMD-derived trend types, rather than to all four original EEMD classes or to the direct magnitude of GPP change.

The collinearity diagnosis confirmed strong correlations among several climatic and hydrometeorological variables. For example, Pearson correlations were high between TEM_mean and PRS_mean (*r* = 0.979), SPEI01_trend and SPEI03_trend (*r* = 0.958), SPEI03_trend and SPEI12_trend (*r* = 0.933), PET_trend and RHU_trend (*r* = −0.917), and PRE_trend and SPEI12_trend (*r* = 0.834). Similar patterns were observed in the Spearman correlation matrix. The VIF analysis also indicated strong multicollinearity, especially for TEM_mean, PET_mean, PRS_mean, RHU_mean, SPEI12_trend, and PET_trend. Therefore, the SHAP importance of individual climatic variables should be interpreted with caution, as correlated predictors may share or redistribute importance within the LightGBM model.

The sensitivity analysis showed that removing SPEI variables changed the rankings of individual climatic predictors, with RHU_mean, SUN_mean, WIND_mean, PRE_trend, and PRE_mean becoming more prominent. When PRE and PET were excluded, SPEI12_mean remained the most important variable, followed by RHU_mean and SPEI12_trend. These results suggest that the exact ranking of individual correlated variables is affected by collinearity. However, the overall conclusion that drought/hydrothermal conditions are dominant controls of nonlinear GPP trend types remains generally robust.

A land cover-specific SHAP summary was further used to examine whether the dominant drivers varied among vegetation types. For forests, RHU_mean and SPEI12_mean were the two most important factors, followed by SUN_mean, SPEI12_trend, and TEM_trend, suggesting that nonlinear trends in forest GPP were closely associated with atmospheric moisture conditions and long-term drought. For cropland, RHU_mean and SPEI12_mean also ranked highly, but TEM_trend, POP_trend, and POP_mean became more prominent, indicating that cropland GPP trends were more strongly associated with both hydrothermal conditions and human activity intensity. In barren and impervious areas, LU- and population-related variables ranked among the most important factors, but these results should be interpreted with caution given the relatively small sample size in this class. Overall, these results suggest that the nonlinear responses of GPP to drought, humidity, precipitation, and human activity are not uniform across land cover types.

Although the total importance of anthropogenic proxies was lower than that of climatic factors, this does not necessarily indicate that human activities were unimportant in the HJRB. The relatively low importance of LU and PD may be partly related to the proxy nature of these variables. LULC and population density primarily capture broad land-cover transformation and population pressure. However, they may not fully represent the timing and intensity of ecological restoration projects, urban expansion, reservoir regulation, or other policy-driven human interventions. Moreover, the continuous decrease type, although small in area, was mainly concentrated around construction and urbanized areas, suggesting that localized negative effects of human activities may be underestimated by basin-wide SHAP importance.

### 3.3 Response of nonlinear GPP variation to driving factors

**Figure 9:**
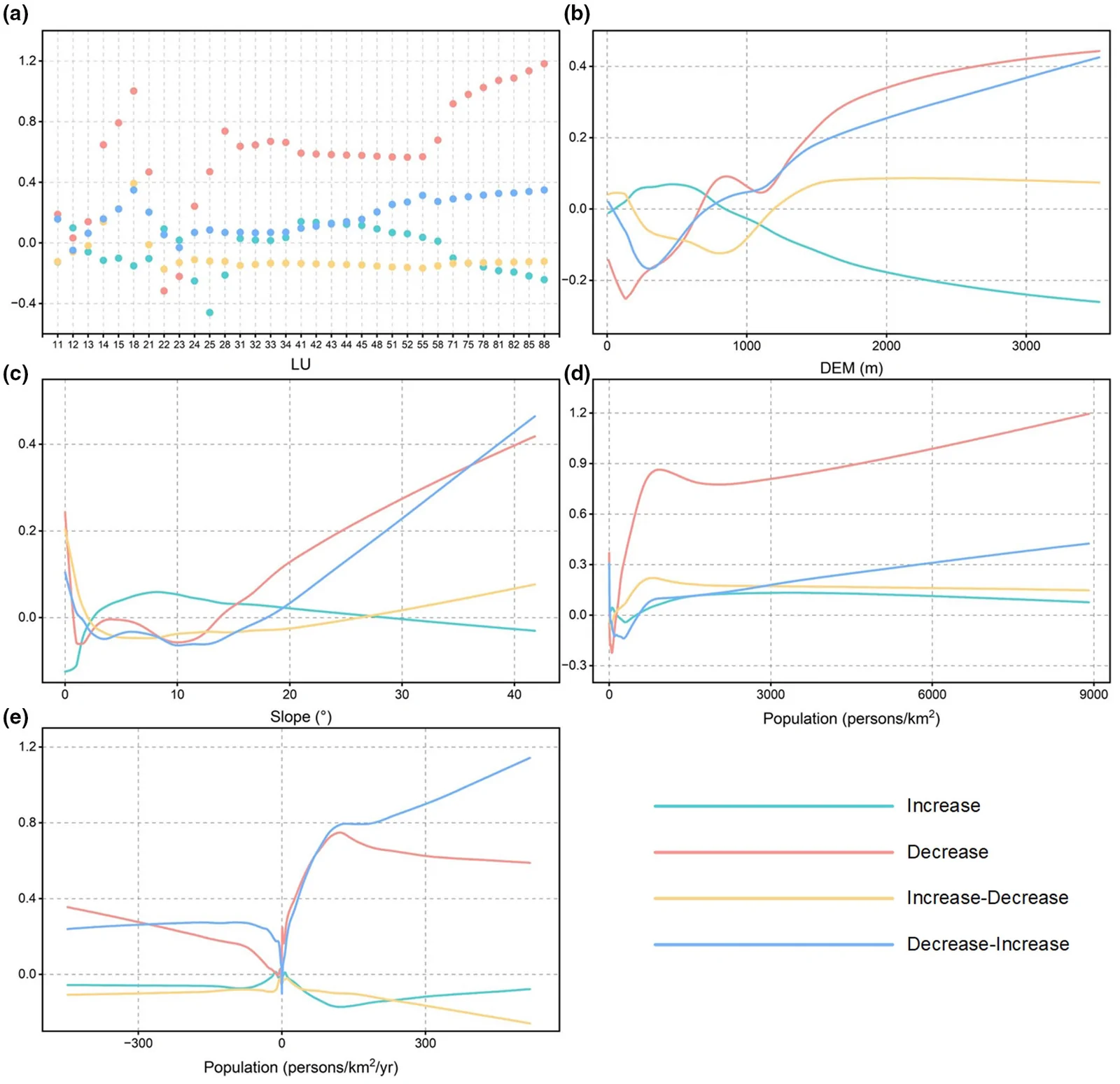
Nonlinear dependence plots of EEMD-derived trends with topographical and anthropogenic factors: **(a)** land use (LU); **(b)** elevation (DEM); **(c)** slope (S); **(d)** population density (PD); and **(e)** population-density trend.

**Figure 10:**
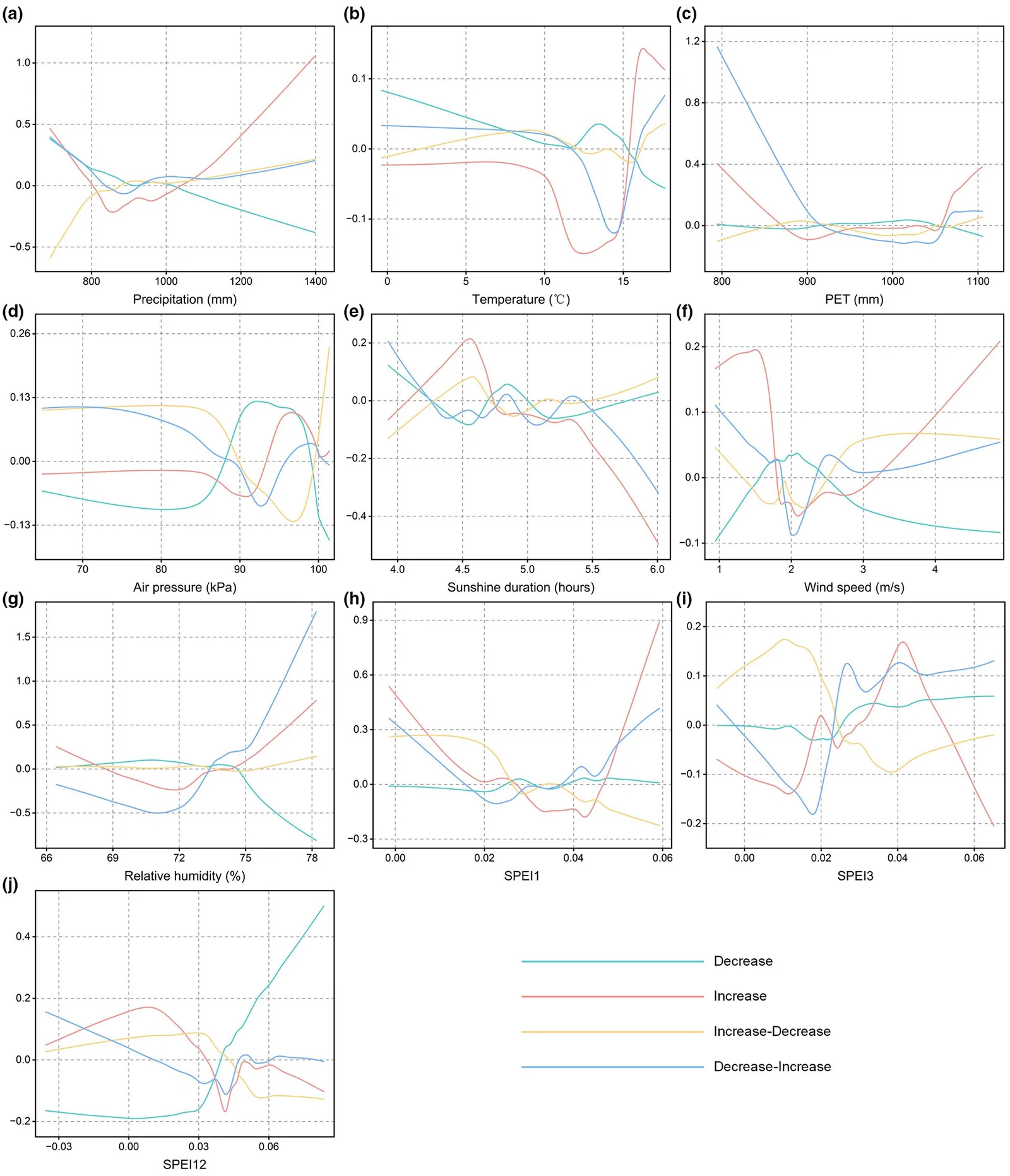
SHAP dependence plots for the EEMD-derived GPP trend types based on annual mean meteorological variables: **(a)** precipitation (PRE); **(b)** temperature (TEM); **(c)** potential evapotranspiration (PET); **(d)** air pressure (PRS); **(e)** sunshine duration (SSD); **(f)** wind speed (WS); **(g)** relative humidity (RHU); **(h)** SPEI1; **(i)** SPEI3; and **(j)** SPEI12.

**Figure 11:**
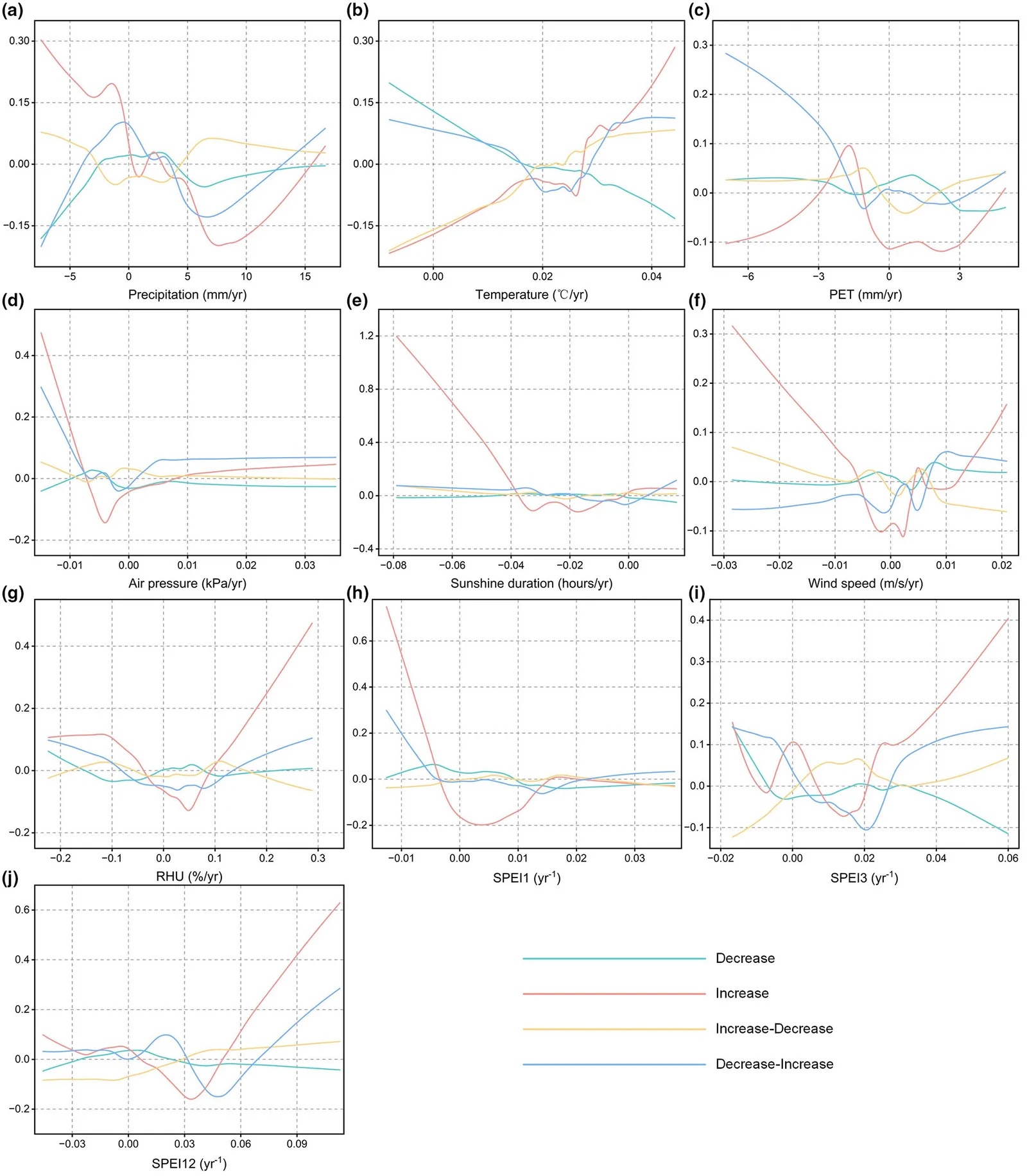
SHAP dependence plots for the EEMD-derived GPP trend types based on temporal trends in meteorological variables: **(a)** PRE trend; **(b)** TEM trend; **(c)** PET trend; **(d)** PRS trend; **(e)** SSD trend; **(f)** WS trend; **(g)** RHU trend; **(h)** SPEI1 trend; **(i)** SPEI3 trend; and **(j)** SPEI12 trend.

The SHAP dependence plots were used to identify how environmental factors altered the SHAP values of different EEMD-derived GPP trend types. Thus, the nonlinear responses described below represent changes in SHAP values for specific GPP trend trajectories, including continuous increase, increase-to-decrease transitions, and decrease-to-increase transitions, rather than direct changes in annual GPP values. This distinction is important because each EEMD trend type reflects a different ecological trajectory: continuous increase indicates sustained improvement; increase-to-decrease indicates emerging constraint or degradation after earlier recovery; decrease-to-increase indicates ecological recovery after an earlier decline; and continuous decrease indicates persistent stress. This study examined the nonlinear dynamics between GPP in the HJRB and its driving factors using nonlinear dependency plots (Figures 9–11), which depict the SHAP values of each factor with respect to GPP trends. Regarding LULC changes, the three trends exhibited limited sensitivity toward variations in LU, except for the transformation of cropland to impervious surfaces (18), which significantly impeded the GPP growth trend. Conversely, the transformation of forests to cropland facilitated the transition of GPP from decline to increase (Figure 9a). As EV increased, the SHAP response of the continuously increasing GPP trend gradually decreased and stabilized after approximately 2,000 m. However, this pattern should be interpreted as an association with elevation-related environmental gradients rather than a direct effect of elevation itself. Higher elevation areas usually differ from low elevation areas in hydrothermal conditions, vegetation composition, soil environment, and human disturbance intensity, which may jointly affect the SHAP responses of different EEMD trend types (Figure 9b). Regarding S variations, the growth and growth to decline trends showed low sensitivity to changes in S, but an increase in S was associated with a higher SHAP response for the transition of GPP from a decline to an increase (Figure 9c). As PD increased, the growth trend of GPP experienced a slight decline, whereas the trend from decline to growth exhibited an upward trajectory (Figure 9d). When the PD change trend was positive, the transition from decreasing to increasing GPP rose rapidly, whereas the increasing trend declined swiftly, with the SHAP value decreasing after peaking. Conversely, when the PD change trend was negative, the SHAP values for all three trend types remained low (Figure 9e).

When PRE was below 800 mm, the SHAP values of all three trend types remained relatively low. Notably, the SHAP dependence plots describe annual-scale associations between climatic variables and EEMD-derived GPP trend types. Therefore, the thresholds identified here should be interpreted as statistical response ranges at the annual scale rather than direct physiological thresholds for short-term climate events. As PRE increased, the SHAP values of the increasing trend tended to decrease, whereas the probabilities of the two transitional trend types increased (Figure 10a). This pattern indicates a nonlinear association between annual precipitation and EEMD-derived GPP trend types. However, it should not be interpreted as a simple causal inhibition effect of precipitation alone. Temperature (TEM) showed no sensitivity to the three trends; however, once TEM exceeded 15 °C, the SHAP value of GPP transitioning from a decline to an increase surged rapidly (Figure 10b). The growth trend exhibited no sensitivity to PET variations, while at approximately 900 mm, the PET both promoted and inhibited the trend of GPP transitioning from a decline to an increase (Figure 10c). Lower values of PRS facilitated the transition of GPP from a decline to an increase but showed no sensitivity to the other trends (Figure 10d). SSD and WS had no significant impact on GPP trend variations, while relative humidity affected only the trend from a decline to an increase to a great extent. Increasing humidity promoted the transition of GPP from decreasing to increasing (Figures 10e–g). Regarding the drought indices (SPEI1, SPEI3, and SPEI12), GPP variations showed no sensitivity to SPEI1 and SPEI3. However, an increase in SPEI12 promoted both the growth trend and the transition from decline to increase in GPP, while inhibiting the transition from increasing to decreasing trend.

The SHAP dependence plots of climatic trends showed clear class-specific nonlinear responses among the three major EEMD-derived GPP trend types (Figure 11). For PRE, TEM, PET, PRS, SSD, and WS trends, the SHAP responses varied across trend classes, indicating that changes in these climatic factors contributed differently to the model prediction of continuous increase, increase-to-decrease, and decrease-to-increase trajectories (Figures 11a–f). However, these responses should be interpreted as statistical associations captured by the LightGBM-SHAP model rather than direct causal effects.

Among the moisture-related variables, RHU showed a relatively distinct response pattern. Increasing RHU was associated with higher SHAP values for the decrease-to-increase trend, suggesting that improved atmospheric moisture conditions may be important for vegetation recovery after an earlier decline (Figure 11g). Compared with precipitation amount or SPEI alone, RHU may more directly reflect near-surface atmospheric water demand and vegetation water stress.

The drought-related indicators also showed scale-dependent SHAP responses. SPEI1 and SPEI3 exhibited relatively weaker or more variable contributions, whereas SPEI12 showed a clearer association with long-term GPP trend differentiation (Figures 11h–j). Because SPEI12 is closely related to PRE, PET, RHU, and other hydrothermal variables, its SHAP response should be interpreted as a long-term drought or wetness association rather than an independent causal effect. Overall, Figure 11 indicates that climatic trends influenced nonlinear GPP trajectories via combined hydrothermal conditions rather than a single climatic factor.

## 4 Discussion

### 4.1 Assessment of the GPP trends using different methods

In examining GPP trends in the HJRB, this study utilized the Theil-Sen estimator and EEMD methods. The findings indicated a significant overall increase in GPP in the HJRB since the onset of the 21st century (Figure 6). This result is consistent with previous studies on vegetation change trends in central China that used linear regression. Additionally, the areas with decreasing trends were concentrated primarily in the urban clusters of the HJRB and its surrounding areas (45, 68, 69). These trends are attributed to alterations in hydrothermal conditions driven by global warming and a series of initiatives implemented in the HJRB over recent decades. These initiatives include the conversion of farmland to forest, afforestation efforts, the establishment of the Danjiangkou Reservoir water conservation and ecological protection areas, and agricultural development in the Jianghan Plain (58, 68, 70, 71). Contrary to studies focusing on the NDVI and vegetation cover (VC), the upstream southern and northern source regions of the HJRB exhibited slower GPP growth trends. Previous studies, including those within the HJRB, primarily focused on detecting linear trends in vegetation change, which imposes significant limitations in terms of revealing the entire natural evolution process of vegetation patterns, since the detected vegetation trend changes represent only the average state over the entire time series (20). Conversely, EEMD effectively mitigates noise and interference from seasonal trends in GPP time series, thereby offering a more accurate depiction of the nonlinear changes in vegetation indices (18, 20, 25, 72). The EEMD analysis in this study revealed nonlinear and gradual changes in GPP trends within the HJRB. From 2000 to 2020, the widespread increase in GPP in the eastern downstream, southern upstream, and central northern source regions of the HJRB gradually transitioned to declining trends, consistent with global studies on vegetation change trends (25). Overall, the GPP in the HJRB region exhibited a stable improving trend while also demonstrating significant nonlinear tendencies.

### 4.2 Quantifying the importance of driving factors in EEMD trends of GPP

The type, distribution, and growth status of vegetation are influenced by a myriad of interacting factors, with climatic conditions being a primary determinant of vegetation growth. Furthermore, anthropogenic activities can exert both direct and indirect impacts on vegetation (73, 74). Factors such as topography and natural disasters can have long-term effects on vegetation types, growth processes, and diversity (75, 76). Previous studies have indicated that climate conditions and human activities are the main driving forces affecting vegetation change, and they are often represented by average temperature and precipitation for climate change and land use change for human activities; however, other important factors that influence vegetation change are overlooked (37, 77, 78, 111). Furthermore, the quantification of driving factors for vegetation change frequently employs the Residual Trends (RESTREND) analysis method to develop linear models. The RESTREND method is straightforward to construct, demonstrates robust performance with smoothed data, and has a wide range of applications. This method enables the quantification of the effects of continuous factors under long-term trends (37, 78, 79).

However, RESTREND fails to accurately capture seasonal changes in data with complex seasonal patterns or non-periodic seasonality. Furthermore, because it relies on linear model construction, RES TREND cannot accurately decompose the trend component of time series data with nonlinear trends (80, 81). Therefore, a careful consideration of the driving factors of vegetation change, while quantifying their importance in nonlinear vegetation dynamics, can reveal the connection between vegetation and its driving factors from a broader perspective. SHAP is based on the concept of Shapley values from cooperative game theory, ensuring fair and reasonable assessment of feature contributions while providing interpretability of model predictions. It can also explain the influence of each feature on the prediction of individual samples (33, 82). Additionally, LightGBM employs decision trees as its base learners. Decision trees are nonlinear models that learn complex decision boundaries through recursive feature selection and node splitting, thereby capturing the intricate nonlinear relationships in the data (28).

The linkage between EEMD trend types and environmental factors can be understood as a trajectory-specific response mechanism. The continuous increase type mainly reflects areas where hydrothermal conditions and ecological restoration jointly supported sustained improvements in productivity. The increase-to-decrease type indicates that earlier vegetation recovery may have become constrained by increasing water stress, excessive precipitation, topographic limitations, or intensified human disturbance. Conversely, the decrease-to-increase type represents a recovery pathway, in which improved humidity conditions, reduced drought stress, or ecological restoration may have reversed earlier declines. Therefore, the LightGBM SHAP results do not merely rank driving factors; rather, they help distinguish which factors are associated with the formation of different nonlinear GPP trajectories. The findings indicated that climatic factors were the predominant drivers of nonlinear changes in vegetation, contributing to 81.71% of the total SHAP-based relative importance. The relatively low importance of LU and PD does not necessarily indicate that regional human activities were unimportant. Similar basin-scale evidence from the Poyang Lake Basin showed that climatic factors had a greater overall effect on vegetation change, whereas human activities were more strongly associated with areas experiencing significant vegetation changes (106). Instead, it suggests that the selected human activity indicators captured only part of the anthropogenic influence. Large-scale ecological restoration programs, such as the Grain for Green/Grass Project, may improve vegetation productivity primarily by converting land cover from cropland or barren land to forest or grassland. However, they may also alter evapotranspiration, water yield, and the carbon-water tradeoff (105–107). Similarly, the South-to-North Water Diversion Project and the ecological protection of the Danjiangkou Reservoir may affect vegetation through reservoir conservation, water source protection, land management, and restrictions on development intensity (109). These processes are partly reflected by LULC and population density, but their project-specific effects were not explicitly quantified in the present model. Conversely, anthropogenic activities and topographical features accounted for 8.50% and 9.79% of the variation, respectively (Table 5). Interestingly, LU changes accounted for a mere 1.84% of the variation. It should be noted that the relatively low importance of LU and PD in the LightGBM SHAP results may partly reflect the exclusion of the continuously decreasing trend type from the classification model. The continuously decreasing type occupied a very small area and was therefore excluded to avoid unstable model training; however, this type was spatially associated with construction and urbanized areas. Thus, the negative impacts of urban expansion and impervious surface growth on GPP may be underestimated when only the three major trend types are modeled. Therefore, the low overall importance of LU should not be interpreted as evidence that human activities had little negative effect, but rather that such negative effects were spatially localized and concentrated in the rare continuous areas of decline. This minimal impact can be attributed to the limited scope of land use modifications, which affected only 7.78% of the region within the HJRB from 2000 to 2020. Moreover, a significant proportion (57.53%) of these changes involved converting farmland to forest. Since the extent of significant changes was relatively small, the importance of LU was low. The proportional importance of PD was 7.02%, closely matching the EV of 7.06%. The HJRB is predominantly populated in the downstream region of the Jianghan Plain, and while urban population density has increased rapidly in recent decades, the changes have occurred mainly in urban areas and their surroundings. However, impervious surfaces in the HJRB accounted for only 2.03%, which resulted in a relatively low importance of PD. Although terrain factors had a relatively small overall contribution, EV showed relatively high importance in the continuously increasing GPP trend. However, because EV is a static topographic factor, it should be treated as an integrated background variable rather than a direct driver of dynamic GPP changes. In the HJRB, elevation is closely related to regional differences in temperature, humidity, precipitation redistribution, vegetation type, soil environment, and human disturbance intensity. The relatively high importance of EV in the increasing trend may therefore indicate that areas with favorable elevation-related environmental conditions, such as mountainous and forested regions with lower disturbance intensity, were more likely to maintain continuous increases in GPP. The significant EV difference between the upstream and downstream of the HJRB, with extensive undisturbed high-altitude forests in the upstream region (58), provides relatively natural environmental conditions for high-altitude forests (83). Additionally, the lower temperatures and higher humidity in mid- to high-EV areas increase plant transpiration rates, promoting plant growth (84). The zonal statistics also indicate that nonlinear GPP dynamics differed between the upper and lower reaches. The upper reach was characterized by a continuous increase in GPP, which may be related to its larger proportion of mountainous and forested areas and a relatively lower intensity of direct human disturbance. By contrast, the lower reach had a substantially higher proportion of increase-to-decrease trends, suggesting that GPP growth was more likely to weaken or reverse in areas with more intensive agricultural production, urban expansion, and land use transformation. This pattern is consistent with previous findings in the upstream and downstream areas of Danjiangkou, where climate factors dominated overall vegetation changes, while upstream human activities were mainly related to returning farmland to forest, and downstream human activities were mainly associated with urban expansion and growth in construction land (85). Therefore, although the basin-wide results provide an overall pattern, subbasin statistics further demonstrate that the nonlinear response of GPP is spatially heterogeneous. The land cover-based SHAP summary further supports this spatial heterogeneity. Forests were mainly affected by RHU and SPEI12, indicating the importance of atmospheric moisture and long-term drought stress for forest productivity. Cropland showed relatively stronger contributions from POP_mean and POP_trend, implying that agricultural management, population concentration, and land use intensity may modify the response of GPP to climatic factors. In barren and impervious areas, population-related variables and land cover variables were more prominent, consistent with the concentration of continuous GPP decrease around construction and urbanized areas. These findings indicate that the nonlinear response of GPP to climatic and human activity factors varies among land cover types, although the interpretation for minor land cover classes should be treated cautiously due to their limited sample sizes. In this study, the most important climate factors (>10%) were SPEI12 (11.27%), PRE (10.75%), and RHU (10.69%). Many studies have indicated that temperature and precipitation are key factors influencing vegetation growth (14, 78, 86, 87). The influence of TEM on the GPP trend in the HJRB was relatively minor, accounting for only 8.02%. Previous research has indicated that vegetation enhancement in the upstream high-altitude mountainous regions of the HJRB is primarily driven by climatic factors other than temperature and precipitation (58). In a study investigating the impact of drought events on GPP in the HJRB from 2001 to 2017, a significant correlation between GPP and drought events was found. Western regions experienced higher drought frequency, while the eastern regions had longer average drought duration and higher drought intensity and severity. The temporal trend of the monthly integrated surface drought index (mISDI) constructed in that study was generally consistent with the spatiotemporal distribution trend of the SPEI in this study (88) (Figure 2). Although the annual PRE in the southern upstream and southeastern downstream regions of the HJRB remained at a relatively high level, the trends of drought indices (SPEI1, 3, and 12) in these two regions and the HJRB source area were lower (Figure 2), resulting in a transition from an increasing to a decreasing trend in GPP in these areas (Figure 7). For the transition from a decreasing to an increasing GPP in the HJRB, RHU was the most important driving factor (17.70%). RHU has significant effects on vegetation transpiration regulation, stomatal regulation, pest control, and nutrient absorption (89).

Additionally, recent studies have indicated the importance of vapor pressure deficit (VPD) on vegetation (90–92). Higher RHU and lower TEM reduce VPD, creating a suitable transpiration environment for plants and facilitating water absorption and nutrient transport (89, 91). The greater importance of RHU than PRE or SPEI in the decrease-to-increase trend may be related to the role of atmospheric moisture in regulating vegetation recovery after stress. PRE and SPEI mainly describe water supply or climatic drought at broader temporal scales, whereas RHU more directly reflects near-surface atmospheric water demand. Higher RHU can reduce vapor pressure deficit, alleviate excessive transpiration, maintain leaf water status, and allow stomata to remain open for photosynthesis. These processes are particularly important during the recovery stage, when vegetation shifts from a declining trajectory to renewed growth. Therefore, RHU may better capture the atmospheric moisture conditions required to reverse GPP decline than precipitation amount alone. Compared with PRE and SPEI, RHU may respond more directly to short-term atmospheric dryness experienced by vegetation canopies. In humid monsoon and mountainous regions such as the HJRB, precipitation amount alone does not necessarily indicate the actual atmospheric water stress experienced by plants. By contrast, RHU affects transpiration demand and stomatal regulation more directly, which may explain why RHU showed a stronger association with the decrease-to-increase recovery pathway. In conclusion, the drivers of GPP demonstrated varying degrees of significance in influencing GPP trend changes, with climatic factors being the most critical. Moreover, when examining the impact of climatic factors on GPP, the high correlations and VIF values among hydrometeorological variables indicate that the importance of individual variables should be interpreted with caution. For example, PRE, PET, RHU, and SPEI are physically linked through the regional water balance, and their SHAP values may partly reflect shared hydrothermal information rather than completely independent effects. Therefore, we interpret these variables as a group of hydroclimatic factors. The sensitivity analysis showed that the dominant water-related signal remained generally stable after removing selected correlated variables, although the ranking of individual drought indicators changed. This result suggests that the overall role of drought and moisture conditions is robust, but the contribution of a single drought index should not be overinterpreted. Furthermore, annual aggregation of SPEI1 and SPEI3 may weaken the ecological meaning of short-term or seasonal drought stress. Therefore, the interpretations of SPEI1 and SPEI3 were treated cautiously, while SPEI12 was emphasized as the more suitable indicator for long-term drought effects on annual GPP trends. Future studies using monthly or growing-season GPP data should further examine growing-season SPEI, lagged SPEI, and cumulative drought indicators. It is imperative to account for the interactions among these factors and their synergistic effects on GPP variations.

### 4.3 Strengths, limitations, and future directions

The main strength of this study lies in integrating EEMD, LightGBM, and SHAP to link the extraction of nonlinear temporal trajectories with interpretable attribution. EEMD separates the nonlinear residual trend of GPP from short-term fluctuations, thereby overcoming the limitation of monotonic trend assumptions. LightGBM can capture nonlinear and interactive relationships among climatic, topographic, and anthropogenic variables, while SHAP provides interpretable feature contributions and response patterns for different EEMD-derived trend types. Furthermore, spatial block cross-validation, multicollinearity diagnostics, sensitivity analysis, and zonal statistics were added to improve the robustness and interpretability of the results.

Nevertheless, several methodological uncertainties remain. First, the EEMD results may be affected by parameter settings, including the number of ensembles and the amplitude of added white noise. Although this study adopted commonly used settings based on previous studies, future work should further assess the sensitivity of EEMD trend classification to different parameter combinations. Second, the LightGBM-SHAP results should be interpreted as statistical associations rather than causal mechanisms. Correlated predictors, especially PRE, PET, RHU, and SPEI, may compete for importance in tree-based models, leading to redistribution of SHAP values among related variables. Therefore, the importance of individual climatic variables should be interpreted with caution, and greater emphasis should be placed on groups of variables such as drought and hydrothermal conditions.

Data limitations should also be acknowledged. This study relied on the MOD17A2HGF GPP product, which is based on a light-use-efficiency model and may be affected by uncertainties in meteorological inputs, biome-specific parameters, cloud contamination, and 8-day composite data gaps. Because no eddy-covariance flux tower observations or alternative GPP products such as FLUXCOM were used for independent validation, the results may be influenced by product-specific biases.

Furthermore, human activities were represented mainly by LULC and population density. Although these indicators are spatially explicit and widely used, they cannot fully capture the timing, intensity, and management effects of major ecological restoration projects, water diversion engineering, urban expansion, impervious-surface changes, nighttime light intensity, reservoir regulation, or agricultural management intensity. The resampling of LandScan population data may also smooth population-density gradients at urban-rural interfaces. Moreover, the continuous decreasing GPP trend type was excluded from the LightGBM-SHAP analysis due to its very small sample size. This treatment improved model stability but may underestimate the negative effects of localized human activities, particularly urban expansion and growth in construction land.

Uncertainty may also arise from temporal-scale aggregation. Vegetation productivity can respond to climatic factors through lagged and cumulative processes, especially for precipitation and drought stress (108, 110). Although annual-scale variables were used to match the annual GPP series and EEMD trend classification, this treatment may obscure the effects of monthly or seasonal extreme events, growing-season drought, and lagged water availability. Future studies could incorporate monthly, growing-season, lagged, and cumulative climatic indicators to further clarify the temporal sensitivity of GPP responses to climate variability.

Finally, the generalizability of the identified nonlinear response patterns should be interpreted with caution. The HJRB is a subtropical monsoon water-source basin with specific hydroclimatic conditions, a history of ecological restoration and human activity patterns. Therefore, the thresholds and response patterns identified in this study may not be directly transferable to arid basins, cold regions, or other ecological zones. Large-scale ecological restoration may enhance ecosystem services but can also increase ecosystem water use and intensify conflicts between vegetation water demand and human water demand (93).

### 4.4 Environmental health implications

Although this study did not directly examine epidemiological outcomes, the nonlinear changes in GPP identified here have important environmental health implications for the HJRB as a major water-source basin. Vegetation productivity affects water conservation, soil erosion control, runoff regulation, and microclimatic buffering, all of which are closely linked to water security and population exposure to environmental risks. Areas showing increase-to-decrease or continuous decreases in GPP may indicate weakened ecosystem functions and greater sensitivity to climate stress or human disturbance.

The results therefore provide an environmental exposure perspective for public health. For example, drought-related declines in GPP may be associated with reduced ecosystem resilience and increased water-security pressures, whereas urban and construction-related GPP decreases may reflect intensified human disturbance and localized environmental degradation. These findings can support ecological restoration, watershed management, and climate-adaptation strategies aimed at reducing environmental risks to regional populations.

### 4.5 Exploring the nonlinear response of GPP's EEMD trends to driving factors

The SHAP dependence results revealed class-specific nonlinear associations between driving factors and EEMD-derived GPP trend types in the HJRB. Although the overall importance of human activity and terrain factors was lower than that of climatic factors (Table 6), these factors still showed distinct nonlinear associations among different EEMD-derived GPP trajectory classes. However, different driving factors still exhibited nonlinear responses to GPP trends. As EV increased, climate conditions, soil characteristics, and environmental factors changed, affecting vegetation types, distribution, and growth status. The SHAP dependence results showed that EV had different associations with different EEMD-derived GPP trend types. For the continuous increase type, the SHAP response tended to decrease and then stabilize as EV increased, particularly between 1,000 and 3,000 m. This suggests that sustained GPP increase may be less strongly associated with mid- to high-elevation areas than with low-elevation or reservoir-surrounding areas. Conversely, the decrease-to-increase type showed relatively higher SHAP responses in the 1,000–3,000 m elevation range, indicating that some mid- to high-elevation regions may have recovered after earlier declines. These divergent patterns do not mean that elevation itself directly promotes or suppresses GPP. Rather, EV represents an integrated environmental gradient that includes temperature, relative humidity, vegetation type, soil conditions, terrain constraints, and human disturbance intensity. Therefore, the EV response should be interpreted as a class-specific association between elevation-related environmental conditions and nonlinear GPP trajectories. When the precipitation deficit is high, it inhibits the increasing trend of GPP but promotes transitions from decreasing to increasing GPP trends.

This result is primarily due to the expansion of human activities, such as agricultural and pastoral activities, initially leads to the destruction of vegetation in areas surrounding urban development (14, 79). However, with the implementation of ecological engineering projects and advances in agricultural modernization, the vegetation trends in these areas have gradually improved. In terms of land use, the expansion of impervious surfaces from urbanization may suppress GPP by replacing vegetation cover, fragmenting habitats, and increasing local heat and water stress (37). Although the continuous decreasing trend type was not included in the LightGBM SHAP model due to its very small sample size, its concentration around construction areas indicates that urban expansion may be an important driver of localized GPP degradation. Therefore, the influence of human activities should be interpreted not only from their average SHAP importance across the three major trend types but also from their spatial association with the excluded continuous decreasing areas.

PRE was an important precipitation-related climatic factor associated with nonlinear changes in GPP trends in the HJRB, but higher PRE levels were associated with lower SHAP values for the continuous increase trend in some areas (94). Therefore, in the HJRB, the SHAP dependence pattern suggests that annual PRE around 800 mm was associated with higher SHAP values of the continuous increase trend, whereas areas with annual PRE exceeding approximately 1,000 mm tended to show lower SHAP values of the continuous increase trend. However, this pattern should be interpreted cautiously. An annual precipitation level of 1,000 mm is not necessarily excessive in the subtropical monsoon climate zone, and most mountainous and hilly areas in the middle and upper reaches of the HJRB have relatively good drainage. Thus, the observed negative SHAP response at higher PRE levels is unlikely to be explained solely by soil waterlogging or root hypoxia (95). A more plausible explanation is that high PRE areas are spatially associated with other environmental constraints. In the HJRB, areas with higher precipitation are often located in mountainous upstream regions, where steep terrain, lower temperatures, reduced solar radiation during cloudy and rainy conditions, and a higher risk of soil erosion may jointly limit vegetation productivity. Furthermore, precipitation is correlated with other hydrothermal variables, such as RHU, PET, SPEI, and sunshine duration, so the PRE response curve may partly reflect the combined effects of regional hydrothermal gradients rather than the independent effect of precipitation alone. Therefore, the threshold around 1,000 mm should be interpreted as a model-derived association under specific regional conditions, rather than as evidence that precipitation above this level directly causes waterlogging stress. Additionally, regions with higher PRE in the HJRB are distributed in upstream high-altitude areas, where excessive PRE can increase the risk of soil erosion. Resulting in soil impoverishment and limitations on plant growth (96). However, vegetation responses to precipitation and drought often involve time lags and cumulative effects, and prolonged drought may offset greenness-induced increases in GPP, especially when productivity and greenness respond asynchronously (97). The annual aggregation used in this study may smooth the effects of monthly extreme precipitation, short-term drought, and growing season water stress. Therefore, the identified precipitation and SPEI thresholds should be interpreted as annual-scale nonlinear associations rather than exact thresholds of vegetation physiological response. This temporal scale aggregation may partly underestimate the sensitivity of GPP to short-term extreme climatic disturbances. Long-term drought (SPEI12) can alter the water and thermal conditions for vegetation and affect photosynthesis and metabolic mechanisms (46, 98), making it the most important limiting factor affecting GPP variations in the HJRB. Thus, PRE and SPEI12 describe related but different aspects of water availability: PRE represents precipitation supply, whereas SPEI12 integrates the long-term balance between precipitation and potential evapotranspiration. The interpretation of the SPEI12 trend should be treated cautiously because SPEI12 is closely related to PRE and PET and may also covary with RHU, sunshine duration, and elevation. Therefore, the positive SHAP response of the increase-to-decrease type at higher SPEI12 trends does not necessarily mean that long-term wetting directly causes vegetation degradation. A more plausible interpretation is that in some parts of the HJRB, increasing wetness may coincide with cloudy and rainy conditions, reduced radiation, steep terrain, or enhanced soil erosion risk, which could weaken the sustained increase in GPP. Thus, the SPEI12 response should be interpreted as a data-driven nonlinear association rather than a simple physiological threshold. The upstream drought conditions in the HJRB significantly improved from 2000 to 2020, while the downstream SPEI exhibited a widespread decrease (Figure 2). Furthermore, research has indicated a significant correlation between drought events in the HJRB and GPP (88). In the HJRB, the influence of TEM on GPP trends mainly manifested through the impact of TEM trends. Suitable TEM increases can enhance the photosynthetic rate of vegetation (Figure 11). However, rapid TEM increases and extremely high TEM can increase plant transpiration rates, leading to drought risks and heat stress that affect plant growth and development (99, 100). PRS in the downstream HJRB significantly decreased over the past 21 years (Figure 2). Although the influence of PRS on vegetation is relatively small, a lower PRS can promote stomatal opening and increase transpiration rates, thereby facilitating vegetation growth (101). In the HJRB, solar radiation has a significant impact on vegetation. Sufficient SSD contributes to the efficiency and progress of photosynthesis in plants (102, 103) while inhibiting the transition from increasing to decreasing GPP trends. In summary, the use of the LightGBM model and SHAP clearly revealed the nonlinear relationships between GPP trend changes in the HJRB and a range of driving factors. This nonlinear trend analysis is particularly necessary for elucidating the response patterns of GPP.

## 5 Conclusions

This study employed EEMD, Theil-Sen trend analysis, and the LightGBM model to analyze and quantify the influence of various driving factors on GPP in the HJRB from 2000 to 2020. The objective was to elucidate the nonlinear response patterns of these driving factors on vegetation productivity. The key findings are as follows:

(1) Over the past 21 years, the OLSR, Theil Sen trend analysis, and EEMD methods all indicated a gradual increase in vegetation productivity in the HJRB. However, the EEMD trend suggested a deceleration in the growth rate of GPP, with significant transitions from increasing to decreasing trends observed in the southern upstream and eastern downstream regions. Conversely, areas with sustained improvements in vegetation productivity were predominantly located in the central and northwest regions of the HJRB.
(2) Results from the LightGBM model and SHAP analysis showed that climatic factors accounted for 81.71% of the total SHAP-based relative importance for nonlinear vegetation trend classification. Among these climate factors, interannual drought (SPEI12, 11.27%), precipitation (PRE, 10.75%), and relative humidity (RHU, 10.69%) emerged as the most influential.
(3) Finally, the nonlinear response relationship between GPP trend changes in the HJRB and its driving factors was illustrated using SHAP. This result demonstrated that GPP variations exhibited nonlinear responses to their driving factors.

In conclusion, this study utilized the EEMD and LightGBM models combined with trend analysis to reveal the nonlinear response relationships of driving factors influencing GPP variations in the HJRB. The method has demonstrated significant applicability in exploring the driving associations of vegetation changes in the HJRB. The proposed methods in this study provide a new solution for understanding the complex nonlinear spatiotemporal variations in vegetation productivity. From an environmental health perspective, nonlinear vegetation productivity changes in this water-source basin may influence water-quality security, soil and water conservation, urban heat mitigation, and the stability of ecosystem services that benefit local and downstream populations. Urban vegetation can mitigate surface warming and reduce heat-related exposure risks, although greening alone may not fully offset urbanization-induced warming (104). Therefore, monitoring GPP dynamics can provide useful environmental evidence for climate-adaptation planning, watershed management, and public-health risk prevention in water-source regions.

However, the results should be interpreted in light of uncertainties from single-source GPP data, EEMD parameter settings, spatial resampling, correlated predictors, and the proxy representation of human activities. Future studies should integrate multi-source GPP validation, direct anthropogenic indicators, and health-impact pathways to strengthen the environmental-health relevance of vegetation productivity research in water-source basins.

## Data Availability

The original contributions presented in the study are included in the article/Supplementary material, further inquiries can be directed to the corresponding authors.

## References

[1] Campbell JE Berry JA Seibt U Smith SJ Montzka SA Launois T . Large historical growth in global terrestrial gross primary production. Nature. (2017) 544:84–7. doi: 10.1038/nature22030 28382993

[2] Chen Y Feng X Tian H Wu X Gao Z Feng Y . Accelerated increase in vegetation carbon sequestration in China after 2010: a turning point resulting from climate and human interaction. Glob Change Biol. (2021) 27:5848–64. doi: 10.1111/gcb.15854 34416063

[3] Heimann M Reichstein M. Terrestrial ecosystem carbon dynamics and climate feedbacks. Nature. (2008) 451:289–92. doi: 10.1038/nature06591 18202646

[4] McDowell NG Beerling DJ Breshears DD Fisher RA Raffa KF Stitt M. The interdependence of mechanisms underlying climate driven vegetation mortality. Trends Ecol Evol. (2011) 26:523–32. doi: 10.1016/j.tree.2011.06.003 21802765

[5] Zeng Z Piao S Li LZX Zhou L Ciais P Wang T . Climate mitigation from vegetation biophysical feedbacks during the past three decades. Nat Clim Change. (2017) 7:432–6. doi: 10.1038/nclimate3299

[6] Anderegg WRL Trugman AT Badgley G Anderson CM Bartuska A Ciais P . Climate driven risks to the climate mitigation potential of forests. Science. (2020) 368:eaaz7005. doi: 10.1126/science.aaz7005 32554569

[7] Bowman DMJS Kolden CA Abatzoglou JT Johnston FH van der Werf GR Flannigan M. Vegetation fires in the Anthropocene. Nat Rev Earth Environ. (2020) 1:500–15. doi: 10.1038/s43017-020-0085-3

[8] Anav A Friedlingstein P Beer C Ciais P Harper A Jones C . Spatiotemporal patterns of terrestrial gross primary production: a review: GPP spatiotemporal patterns. Rev Geophys. (2015) 53:785–818. doi: 10.1002/2015RG000483

[9] Fu B Wang S Liu Y Liu J Liang W Miao C. Hydrogeomorphic ecosystem responses to natural and anthropogenic changes in the Loess Plateau of China. Annu Rev Earth Planet Sci. (2017) 45:223–43. doi: 10.1146/annurev-earth-063016-020552

[10] Li P Wang J Mengmeng L Xue Z Bagherzadeh A Mengyun L. Spatio temporal variation characteristics of NDVI and its response to climate on the Loess Plateau from 1985 to 2015. CATENA. (2021) 203:105331. doi: 10.1016/j.catena.2021.105331

[11] Feng X Fu B Zhang Y Pan N Zeng Z Tian H . Recent leveling off of vegetation greenness and primary production reveals the increasing soil water limitations on the greening Earth. Sci Bull. (2021) 66:1462–71. doi: 10.1016/j.scib.2021.02.023 36654372

[12] Zhang Z Jin G. Spatiotemporal differentiation of carbon budget and balance zoning: insights from the middle reaches of the Yangtze River Urban Agglomeration, China. Appl Geograp. (2024) 167:103293. doi: 10.1016/j.apgeog.2024.103293

[13] Erb KH Kastner T Plutzar C Bais ALS Carvalhais N Fetzel T . Unexpectedly large impact of forest management and grazing on global vegetation biomass. Nature. (2018) 553:73–6. doi: 10.1038/nature25138 29258288 PMC5756473

[14] Wei Y Lu H Wang JN Wang X Sun J. Dual influence of climate change and anthropogenic activities on the spatiotemporal vegetation dynamics over the Qinghai-Tibetan Plateau from 1981 to 2015. Earth's Future. (2022) 10. doi: 10.1029/2021EF002566

[15] Zheng L Zhao G Dong J Ge Q Tao J Zhang X . Spatial, temporal, and spectral variations in albedo due to vegetation changes in China's grasslands. ISPRS J Photogramm Remote Sens. (2019) 152:1–12. doi: 10.1016/j.isprsjprs.2019.03.020

[16] Fang X Zhu Q Ren L Chen H Wang K Peng C. Large scale detection of vegetation dynamics and their potential drivers using MODIS images and BFAST: a case study in Quebec, Canada. Remote Sens Environ. (2018) 206:391–402. doi: 10.1016/j.rse.2017.11.017

[17] Atkinson PM Jeganathan C Dash J Atzberger C. Inter comparison of four models for smoothing satellite sensor time series data to estimate vegetation phenology. Remote Sens Environ. (2012) 123:400–17. doi: 10.1016/j.rse.2012.04.001

[18] Hawinkel P Swinnen E Lhermitte S Verbist B Van Orshoven J Muys B. A time series processing tool to extract climate driven interannual vegetation dynamics using Ensemble Empirical Mode Decomposition (EEMD). Remote Sens Environ. (2015) 169:375–89. doi: 10.1016/j.rse.2015.08.024

[19] Liu YY van Dijk AIJM McCabe MF Evans JP de Jeu RAM. Global vegetation biomass change (1988 2008) and attribution to environmental and human drivers: global vegetation biomass change. Glob Ecol Biogeogr. (2013) 22:692–705. doi: 10.1111/geb.12024

[20] Yang L Guan Q Lin J Tian J Tan Z Li H. Evolution of NDVI secular trends and responses to climate change: a perspective from nonlinearity and nonstationarity characteristics. Remote Sens Environ. (2021) 254:112247. doi: 10.1016/j.rse.2020.112247

[21] Zhou Z Liu S Ding Y Fu Q Wang Y Cai H . Assessing the responses of vegetation to me teorological drought and its influencing factors with partial wavelet coherence analysis. J Environ Manag. (2022) 311:114879. doi: 10.1016/j.jenvman.2022.114879 35303597

[22] Wu Z Huang NE. Ensemble empirical mode decomposition: a noise assisted data analysis method. Adv Adapt Data Anal. (2009) 1:1–41. doi: 10.1142/S1793536909000047

[23] Huang NE Shen Z Long SR Wu MC Shih HH Zheng Q . The empirical mode decomposition and the Hilbert spectrum for nonlinear and nonstationary time series analysis. Proc R Soc Lond A. (1998) 454:903–95. doi: 10.1098/rspa.1998.0193

[24] Liu K Li X Wang S Zhang X. Unrevealing past and future vegetation restoration on the Loess Plateau and its impact on terrestrial water storage. J Hydrol. (2023) 617:129021. doi: 10.1016/j.jhydrol.2022.129021

[25] Pan N Feng X Fu B Wang S Ji F Pan S. Increasing global vegetation browning hidden in overall vegetation greening: insights from time varying trends. Remote Sens Environ. (2018) 214:59–72. doi: 10.1016/j.rse.2018.05.018

[26] Wu Z Huang NE Long SR Peng CK. On the trend, detrending, and variability of nonlinear and nonstationary time series. Proc Natl Acad Sci USA (2007) 104:14889–94. doi: 10.1073/pnas.0701020104 17846430 PMC1986583

[27] Xiao X Wang Q Guan Q Zhang Z Yan Y Mi J . Quantifying the nonlinear response of vegetation greening to driving factors in Longnan of China based on machine learning algorithm. Ecol Indic. (2023) 151:110277. doi: 10.1016/j.ecolind.2023.110277

[28] Ke G Meng Q Finley T Wang T Chen W Ma W . LightGBM: a highly efficient gradient boosting decision tree. In:Guyon I Luxburg UV Bengio S Wallach H Fergus R Vishwanathan S et al., editors. Advances in Neural Information Processing Systems, vol. 30. Red Hook, NY: Curran Associates, Inc. (2017). p. 3146–54.

[29] Chen T Guestrin C. XGBoost: a scalable tree boosting system. In: Proceedings of the 22nd ACM SIGKDD international conference on knowledge discovery and data mining. New York, NY: Association for Computing Machinery (ACM) (2016). p. 785–94. doi: 10.1145/2939672.2939785

[30] Chen H Huang S Xu YP Teegavarapu RSV Guo Y Nie H . River ecological flow early warning forecasting using baseflow separation and machine learning in the Jiaojiang River Basin, Southeast China. Sci Total Environ. (2023) 882:163571. doi: 10.1016/j.scitotenv.2023.163571 37087001

[31] Guo X Gui X Xiong H Hu X Li Y Cui H . Critical role of climate factors for groundwater potential mapping in arid regions: insights from random forest, XGBoost, and LightGBM algorithms. J Hydrol. (2023) 621:129599. doi: 10.1016/j.jhydrol.2023.129599

[32] Li B Liu K Wang M Wang Y He Q Zhuang L . High spatiotemporal resolution dynamic water monitoring using LightGBM model and Sentinel 2 MSI data. Int J Appl Earth Obs Geoinf. (2023) 118:103278. doi: 10.1016/j.jag.2023.103278

[33] Wang D Thunéll S Lindberg U Jiang L Trygg J Tysklind M. Towards better process management in wastewater treatment plants: process analytics based on SHAP values for tree based machine learning methods. J Environ Manag. (2022) 301:113941. doi: 10.1016/j.jenvman.2021.113941 34731954

[34] Wei C Wu Z Xing J Jin G. Trade off or synergy? Dynamic analysis and policy insights on land use functions in China. Environ Impact Assess Rev. (2024) 105:107399. doi: 10.1016/j.eiar.2023.107399

[35] Zheng L Lu J Liu H Chen X Yesou H. Evidence of vegetation greening benefitting from the afforestation initiatives in China. Geo Spat Inf Sci. (2024) 27:683–702. doi: 10.1080/10095020.2023.2238782

[36] Cong N Wang T Nan H Ma Y Wang X Myneni RB . Changes in satellite derived spring vegetation green up date and its linkage to climate in China from 1982 to 2010: a multimethod analysis. Glob Change Biol. (2013) 19:881–91. doi: 10.1111/gcb.12077 23504844

[37] Ge W. Quantifying the contributions of human activities and climate change to vegetation net primary productivity dynamics in China from 2001 to 2016. Sci Total Environ. (2021) 11. doi: 10.1016/j.scitotenv.2021.145648 33582337

[38] Tong X Wang K Yue Y Brandt M Liu B Zhang C . Quantifying the effectiveness of ecological restoration projects on long term vegetation dynamics in the karst regions of Southwest China. Int J Appl Earth Obs Geoinf. (2017) 54:105–13. doi: 10.1016/j.jag.2016.09.013

[39] Vicente Serrano SM Beguería S Lorenzo Lacruz J Camarero JJ López Moreno JI Azorin Molina C . Performance of drought indices for ecological, agricultural, and hydrological applications. Earth Interact. (2012) 16:1–27. doi: 10.1175/2012EI000434.1

[40] Gupta A Rico Medina A Caño Delgado AI. The physiology of plant responses to drought. Science. (2020) 368:266–9. doi: 10.1126/science.aaz7614 32299946

[41] Mishra AK Singh VP. A review of drought concepts. J Hydrol. (2010) 391:202–16. doi: 10.1016/j.jhydrol.2010.07.012

[42] Chen J Shao Z Huang X Zhuang Q Dang C Cai B . Assessing the impact of drought land cover change on global vegetation greenness and productivity. Sci Total Environ. (2022) 852:158499. doi: 10.1016/j.scitotenv.2022.158499 36058327

[43] Zhang Q Kong D Singh VP Shi P. Response of vegetation to different time scales drought across China: spatiotemporal patterns, causes and implications. Glob Planet Change. (2017) 152:1–11. doi: 10.1016/j.gloplacha.2017.02.008

[44] Um MJ Kim Y Jung K Lee M An H Min I . Evaluation of drought propagations with multiple indices in the Yangtze River basin. J Environ Manag. (2022) 317:115494. doi: 10.1016/j.jenvman.2022.115494 35751287

[45] Zhang J Zhang Y Sun G Song C Li J Hao L . Climate variability masked greening effects on water yield in the Yangtze River Basin During 2001–2018. Water Resour Res. (2022) 58:e2021WR030382. doi: 10.1029/2021WR030382

[46] Zhou L Zhang X Zhang Y Song Q Myo STZ Zhou R . The cumulative drought exert disruptive effects on tropical rainforests in the northern edge of Asia based on decadal dendrometric measurements and eddy covariance method. Agric For Meteorol. (2022) 316:108858. doi: 10.1016/j.agrformet.2022.108858

[47] Mottl O Flantua SGA Bhatta KP Felde VA Giesecke T Goring S . Global acceleration in rates of vegetati on change over the past 18,000 years. Science. (2021) 372:860–4. doi: 10.1126/science.abg1685 34016781

[48] Gu Z Duan X Shi Y Li Y Pan X. Spatiotemporal variation in vegetation coverage and its response to climatic factors in the Red River Basin, China. Ecol Indic. (2018) 93:54–64. doi: 10.1016/j.ecolind.2018.04.033

[49] Li S Liang W Fu B Lü Y Fu S Wang S . Vegetation changes in recent large scale ecological restoration projects and subsequent impact on water resources in China's Loess Plateau. Sci Total Environ. (2016) 569–570:1032–9. doi: 10.1016/j.scitotenv.2016.06.141 27387800

[50] Zhao H Wu C Wang X. Large scale forest conservation and restoration programs significantly contributed to land surface greening in China. Environ Res Lett. (2022) 17:024023. doi: 10.1088/1748-9326/ac44c5

[51] Wang J Lu J Zheng L Zhang Z Chen X. Impacts of land use change and vegetation greening on gross primary productivity in the Yangtze River Basin from a water-carbon coupling perspective. GISci Remote Sens. (2025) 62:2578033. doi: 10.1080/15481603.2025.2578033

[52] Zhou Y Guo S Hong X Chang FJ. Systematic impact assessment on inter basin water transfer projects of the Hanjiang River Basin in China. J Hydrol. (2017) 553:584–95. doi: 10.1016/j.jhydrol.2017.08.039

[53] Schwalm CR Anderegg WRL Michalak AM Fisher JB Biondi F Koch G . Global patterns of drought recovery. Nature. (2017) 548:202–5. doi: 10.1038/nature23021 28796213

[54] Allen R. An update for the calculation of reference evapotranspiration. IcID Bull. (1994) 43:35–92.

[55] Vicente Serrano SM Beguería S López Moreno JI. A multiscalar drought index sensitive to global warming: the standardized precipitation evapotranspiration index. J Clim. (2010) 23:1696–718. doi: 10.1175/2009JCLI2909.1

[56] Hutchinson MF. ANUSPLIN Version 4.2 User Guide. Canberra, ACT: Centre for Resource and Environmental Studies, The Australian National University (2004). p. 54.

[57] Guo P Zhang F Wang H. The response of ecosystem service value to land use change in the middle and lower Yellow River: a case study of the Henan section. Ecol Indic. (2022) 140:109019. doi: 10.1016/j.ecolind.2022.109019

[58] Yang S Liu J Wang C Zhang T Dong X Liu Y. Vegetation dynamics influenced by climate change and human activities in the Hanjiang River Basin, central China. Ecol Indic. (2022) 145:109586. doi: 10.1016/j.ecolind.2022.109586

[59] Yang J Huang X. The 30 mannual land cover dataset and its dynamics in China from 1990 to 2019. Earth Syst Sci Data. (2021) 13:3907–25. doi: 10.5194/essd-13-3907-2021

[60] Weber M Moehl J Weston S Rose A Brelsford C Hauser T. LandScan USA 2021. Oak Ridge, TN: Oak Ridge National Laboratory (ORNL) (2021).

[61] Sen PK. Estimates of the regression coefficient based on Kendall's Tau. J Am Stat Assoc. (1968) 63:1379–89. doi: 10.1080/01621459.1968.10480934

[62] Fernandes R Leblanc SG. Parametric (modified least squares) and nonparametric (Theil–Sen) linear regressions for predicting biophysical parameters in the presence of measurement errors. Remote Sens Environ. (2005) 95:303–16. doi: 10.1016/j.rse.2005.01.005

[63] Mann HB. Nonparametric tests against trend. Econometrica. (1945) 13:245. doi: 10.2307/1907187

[64] Feurer M Hutter F Kotthoff L Vanschoren J. Automated Machine Learning: Methods, Systems, Challenges. Cham: Springer (2019). doi: 10.1007/978-3-030-05318-5

[65] Snoek J Larochelle H Adams RP. Practical Bayesian optimization of machine learning algorithms. In:Pereira F Burges CJC Bottou L Weinberger KQ, editors. Advances in Neural Information Processing Systems (NeurIPS 2012), vol. 25. Red Hook, NY: Curran Associates, Inc. (2012). p. 2951–9.

[66] Wu J Chen X Zhang H Xiong LD Lei H Deng S. Hyperparameter optimization for machine learning models based on Bayesian optimization. J Electron Sci Technol. (2019) 17:26–40. doi: 10.11989/jest.1674-862x.80904120

[67] Cleveland WS. Robust locally weighted regression and smoothing scatterplots. J Am Stat Assoc. (1979) 74:829–36. doi: 10.1080/01621459.1979.10481038

[68] Chen T Xia J Zou L Hong S. Quantifying the influences of natural factors and human activities on NDVI changes in the Hanjiang River Basin, China. Remote Sens. (2020) 12:3780. doi: 10.3390/rs12223780

[69] Liu H Zheng L Yin S. Multi perspective analysis of vegetation cover changes and driving factors of long time series based on climate and terrain data in Hanjiang River Basin, China. Arab J Geosci. (2018) 11:509. doi: 10.1007/s12517-018-3756-3

[70] Tong Q Swallow B Zhang L Zhang J. The roles of risk aversion and climate smart agriculture in climate risk management: evidence from rice production in the Jianghan Plain, China. Clim Risk Manag. (2019) 26:100199. doi: 10.1016/j.crm.2019.100199

[71] Zhu Z Piao S Myneni RB Huang M Zeng Z Canadell JG . Greening of the Earth and its drivers. Nat Clim Change. (2016) 6:791–5. doi: 10.1038/nclimate3004

[72] Jong R Verbesselt J Schaepman ME Bruin S. Trend changes in global greening and browning: contribution of short term trends to longer term change. Glob Change Biol. (2012) 18:642–55. doi: 10.1111/j.1365-2486.2011.02578.x

[73] De Jong R Schaepman ME Furrer R De Bruin S Verburg PH. Spatial relationship between climatologies and changes in global vegetation activity. Glob Change Biol. (2013) 19:1953–64. doi: 10.1111/gcb.12193 23512439

[74] Wang L Tian F Wang Y Wu Z Schurgers G Fensholt R. Acceleration of global vegetation greenup from combined effects of climate change and human land management. Glob Change Biol. (2018) 24:5484–99. doi: 10.1111/gcb.14369 29963745

[75] Allen CD Macalady AK Chenchouni H Bachelet D McDowell N Vennetier M . A global overview of drought and heat induced tree mortality reveals emerging climate change risks for forests. For Ecol Manag. (2010) 259:660–84. doi: 10.1016/j.foreco.2009.09.001

[76] Sitch S Friedlingstein P Gruber N Jones SD Murray Tortarolo G Ahlström A . Recent trends and drivers of regional sources and sinks of carbon dioxide. Biogeosciences. (2015) 12:653–79. doi: 10.5194/bg-12-653-2015

[77] Gao W Zheng C Liu X Lu Y Chen Y Wei Y . NDVI based vegetation dynamics and their responses to climate change and human activities from 1982 to 2020: a case study in the Mu Us Sandy Land, China. Ecol Indic. (2022) 137:108745. doi: 10.1016/j.ecolind.2022.108745

[78] Ma M Wang Q Liu R Zhao Y Zhang D. Effects of climate change and human activities on vegetation coverage change in northern China considering extreme climate and time lag and accumulation effects. Sci Total Environ. (2023) 860:160527. doi: 10.1016/j.scitotenv.2022.160527 36460108

[79] Ren Z Tian Z Wei H Liu Y Yu Y. Spatiotemporal evolution and driving mechanisms of vegetation in the Yellow River Basin, China during 2000–2020. Ecol Indic. (2022) 138:108832. doi: 10.1016/j.ecolind.2022.108832

[80] Burrell AL Evans JP Liu Y. Detecting dryland degradation using Time Series Segmentation and Residual Trend analysis (TSS RESTREND). Remote Sens Environ. (2017) 197:43–57. doi: 10.1016/j.rse.2017.05.018

[81] Huang NE Wu Z. A review on Hilbert Huang transform: Method and its applications to geophysical studies. Rev Geophys. (2008) 46:RG2006. doi: 10.1029/2007RG000228

[82] Lundberg SM Lee SI. A unified approach to interpreting model predictions. In:Guyon I von Luxburg U Bengio S Wallach H Fergus R Vishwanathan S et al., editors. Advances in Neural Information Processing Systems 30. Red Hook, NY: Curran Associates, Inc. (2017). p. 4765–74.

[83] Ioki K James D Phua MH Tsuyuki S Imai N. Recovery of tree community composition across different types of anthropogenic disturbances and characterization of their effect using Landsat time series in Bornean tropical montane forest. Biol Conserv. (2022) 267:109489. doi: 10.1016/j.biocon.2022.109489

[84] Grossiord C Buckley TN Cernusak LA Novick KA Poulter B Siegwolf RTW . Plant responses to rising vapor pressure deficit. New Phytol. (2020) 226:1550–66. doi: 10.1111/nph.16485 32064613

[85] Liu H Liu F Yuan H Zheng L Zhang Y. Assessing the relative role of climate and human activities on vegetation cover changes in the up-down stream of Danjiangkou, China. J Plant Ecol. (2022) 15:180–95. doi: 10.1093/jpe/rtab082

[86] Zheng K Wei JZ Pei JY Cheng H Zhang XL Huang FQ . Impacts of climate change and human activities on grassland vegetation variation in the Chinese Loess Plateau. Sci Total Environ. (2019) 660:236–44. doi: 10.1016/j.scitotenv.2019.01.022 30640092

[87] Zhu L Sun S Li Y Liu X Hu K. Effects of climate change and anthropogenic activity on the vegetation greening in the Liaohe River Basin of northeastern China. Ecol Indic. (2023) 148:110105. doi: 10.1016/j.ecolind.2023.110105

[88] Jiang W Wang L Zhang M Yao R Chen X Gui X . Analysis of drought events and their impacts on vegetation productivity based on the integrated surface drought index in the Hanjiang River Basin, China. Atmos Res. (2021) 254:105536. doi: 10.1016/j.atmosres.2021.105536

[89] Grantz DA. Plant response to atmospheric humidity. Plant Cell Environ. (1990) 13:667–79. doi: 10.1111/j.1365-3040.1990.tb01082.x

[90] Dubey N Ghosh S. The relative role of soil moisture and vapor pressure deficit in affecting the Indian vegetation productivity. Environ Res Lett. (2023) 18:064012. doi: 10.1088/1748-9326/acd2ef

[91] Song Y Jiao W Wang J Wang L. Increased global vegetation productivity despite rising atmospheric dryness over the last two decades. Earth's Future. (2022) 10:e2021EF002634. doi: 10.1029/2021EF002634

[92] Yuan W Zheng Y Piao S Ciais P Lombardozzi D Wang Y . (2019). Increased atmospheric vapor pressure deficit reduces global vegetation growth. Sci Adv. 5:eaax1396. doi: 10.1126/sciadv.aax1396 PMC6693914 31453338

[93] Zhu N Lu J Wang J Zheng L Zhang P Chen X. Current intensified water resource conflicts in China's Loess Plateau may be alleviated under future climate change. Ecohydrology. (2026) 19:e70211. doi: 10.1002/eco.70211

[94] Piao S Yin G Tan J Cheng L Huang M Li Y . Detection and attribution of vegetation greening trend in China over the last 30 years. Glob Change Biol. (2015) 21:1601–9. doi: 10.1111/gcb.12795 25369401

[95] Pan J Sharif R Xu X Chen X. Mechanisms of waterlogging tolerance in plants: research progress and prospects. Front Plant Sci. (2021) 11:627331. doi: 10.3389/fpls.2020.627331 33643336 PMC7902513

[96] Sivakumar MVK Stefanski R. Climate and land degradation—an overview. In:Sivakumar MVK Ndiang'ui N, editors. Climate and Land Degradation. Environmental Science and Engineering. Berlin, Heidelberg: Springer (2007). doi: 10.1007/978-3-540-72438-4_6

[97] Zheng L Lu J Chen X. Drought offsets the vegetation greenness-induced gross primary productivity from 1982 to 2018 in China. J Hydrol. (2024) 632:130881. doi: 10.1016/j.jhydrol.2024.130881

[98] Zhang Z Ju W Zhou Y Li X. Revisiting the cumulative effects of drought on global gross primary productivity based on new long-term series data (1982–2018). Glob Chang Biol. (2022) 28:3620–35. doi: 10.1111/gcb.16178 35343026

[99] Lloret F Escudero A Iriondo JM Martínez-Vilalta J Valladares F. Extreme climatic events and vegetation: the role of stabilizing processes. Glob Change Biol. (2012) 18:797–805. doi: 10.1111/j.1365-2486.2011.02624.x

[100] Luo M Sa C Meng F Duan Y Liu T Bao Y. Assessing extreme climatic changes on a monthly scale and their implications for vegetation in Central Asia. J Clean Prod. (2020) 271:122396. doi: 10.1016/j.jclepro.2020.122396

[101] Daunicht HJ Brinkjans HJ. Plant responses to reduced air pressure: advanced techniques and results. Adv Space Res. (1996) 18:273–81. doi: 10.1016/0273-1177(95)00889-M 11538810

[102] Che M Chen B Innes JL Wang G Dou X Zhou T . Spatial and temporal variations in the end date of the vegetation growing season throughout the Qinghai–Tibetan Plateau from 1982 to 2011. Agric For Meteorol. (2014) 189–190:81–90. doi: 10.1016/j.agrformet.2014.01.004

[103] Hu MQ Mao F Sun H Hou YY. Study of normalized difference vegetation index variation and its correlation with climate factors in the three river source region. Int J Appl Earth Obs Geoinf. (2011) 13:24–33. doi: 10.1016/j.jag.2010.06.003

[104] Liu F Wang L Cao Q Gu X Xiao W Zhang X . Urban greening mitigates surface warming induced by urbanization in China. Agric For Meteorol. (2026) 381:111062. doi: 10.1016/j.agrformet.2026.111062

[105] Chen C Park T Wang X Piao S Xu B Chaturvedi RK . China and India lead in greening of the world through land-use management. Nat Sustain. (2019) 2:122–9. doi: 10.1038/s41893-019-0220-7 30778399 PMC6376198

[106] Liu H Zheng L Liao M. Dynamics of vegetation change and its relationship with nature and human activities — a case study of Poyang Lake Basin, China. J Sustain For. (2021) 40:47–67. doi: 10.1080/10549811.2020.1738947

[107] Ruan L He T Xiao W Chen W Lu D Liu S. Measuring the coupling of builtup land intensity and use efficiency: an example of the Yangtze River Delta urban agglomeration. Sustain Cities Soc. (2022) 87:104224. doi: 10.1016/j.scs.2022.104224

[108] Wu J Gao W Zheng Z Zhao D Zeng Y. Study of human activity intensity from 2015 to 2020 based on remote sensing in Anhui Province, China. Remote Sens. (2023) 15:2029. doi: 10.3390/rs15082029

[109] Zhan Y Fan J Meng T Li Z Yan Y Huang J . Analysis on vegetation cover changes and the driving factors in the mid lower reaches of Hanjiang River Basin between 2001 and 2015. Open Geosci. (2021) 13:675–89. doi: 10.1515/geo-2020-0259

[110] Zheng L Wu H Lu J Wang J Li T Lin A . Increased precipitation partially alleviates the carbon-water tradeoff under the Grain for Green program. Environ Res. (2026) 306:125069. doi: 10.1016/j.envres.2026.125069 42309428

[111] Zheng L Wu H Lin A Lu J Chen X. Climate change weakened the productivity benefits from the forestry ecological engineering projects-induced greening in China. Int J Appl Earth Obs Geoinf. (2026) 146:105052. doi: 10.1016/j.jag.2025.105052

