# Supplementary material for: Nonlinear response of vegetation productivity to climate change and human activities in a water-source basin: implications for environmental health and water security
Source: Front Public Health. 2026 Aug 26;14:1868335. doi: 10.3389/fpubh.2026.1868335 (PMC13553843; doi:10.3389/fpubh.2026.1868335)
Supplement: Supplementary file 1 [file Supplementary_file_1.docx]

Figure S1：Pearson/Spearman correlation matrices

Table S1：VIF results

| variable | VIF |
| --- | --- |
| TEM_mean | 1000.0129 |
| PET_mean | 731.9264 |
| PRS_mean | 588.7775 |
| WIND_mean | 312.0994 |
| SUN_mean | 112.7433 |
| RHU_mean | 94.4956 |
| SPEI12_trend | 68.3482 |
| RHU_trend | 67.2717 |
| PET_trend | 61.7363 |
| PRS_trend | 53.4536 |
| SPEI03_trend | 35.4062 |
| WIND_trend | 34.4184 |
| SUN_trend | 33.2244 |
| SPEI01_trend | 26.0318 |
| PRE_mean | 22.2846 |
| SPEI12_mean | 17.2515 |
| SPEI03_mean | 16.2793 |
| PRE_trend | 15.4887 |
| SPEI01_mean | 15.4165 |
| TEM_trend | 13.2987 |

Table S2：feature-set sensitivity analysis

| feature | SHAP_Full | SHAP_No_SPEI | SHAP_No_PRE_PET | rank_Full | rank_No_SPEI | rank_delta_No_SPEI | rank_No_PRE_PET | rank_delta_No_PRE_PET |
| --- | --- | --- | --- | --- | --- | --- | --- | --- |
| DEM | 0.0558 | 0.0645 | 0.0531 | 22 | 18 | 4 | 19 | 3 |
| LU_is_Barren_Impervious | 0.0025 | 0.0031 | 0.0027 | 27 | 21 | 6 | 23 | 4 |
| LU_is_Cropland | 0.0181 | 0.0217 | 0.0216 | 25 | 19 | 6 | 21 | 4 |
| LU_is_Forest | 0.0109 | 0.0101 | 0.0208 | 26 | 20 | 6 | 22 | 4 |
| LU_is_Grassland | 0.0002 | 0.0000 | 0.0002 | 29 | 26.5 | 2.5 | 25 | 4 |
| LU_is_Shrub | 0.0000 | 0.0000 | 0.0000 | 30 | 26.5 | 3.5 | 28 | 2 |
| LU_is_Water | 0.0016 | 0.0022 | 0.0016 | 28 | 22 | 6 | 24 | 4 |
| PET_mean | 0.0580 | 0.0789 | 0.0000 | 20 | 15 | 5 | 28 | 8 |
| PET_trend | 0.0508 | 0.0896 | 0.0000 | 23 | 14 | 9 | 28 | 5 |
| POP_mean | 0.0880 | 0.0977 | 0.0862 | 9 | 8 | 1 | 10 | 1 |
| POP_trend | 0.1049 | 0.0935 | 0.0963 | 6 | 11 | 5 | 8 | 2 |
| PRE_mean | 0.0936 | 0.1144 | 0.0000 | 8 | 5 | 3 | 28 | 20 |
| PRE_trend | 0.0828 | 0.1214 | 0.0000 | 12 | 4 | 8 | 28 | 16 |
| PRS_mean | 0.0425 | 0.0973 | 0.0666 | 24 | 9 | 15 | 17 | 7 |
| PRS_trend | 0.0591 | 0.0648 | 0.0691 | 19 | 17 | 2 | 16 | 3 |
| RHU_mean | 0.1642 | 0.1961 | 0.2385 | 2 | 1 | 1 | 2 | 0 |
| RHU_trend | 0.0750 | 0.0739 | 0.0795 | 14 | 16 | 2 | 14 | 0 |
| SLOPE | 0.0857 | 0.0916 | 0.0827 | 11 | 13 | 2 | 11 | 0 |
| SPEI01_mean | 0.0672 | 0.0000 | 0.0864 | 15 | 26.5 | 11.5 | 9 | 6 |
| SPEI01_trend | 0.0617 | 0.0000 | 0.0783 | 17 | 26.5 | 9.5 | 15 | 2 |
| SPEI03_mean | 0.1245 | 0.0000 | 0.1188 | 3 | 26.5 | 23.5 | 6 | 3 |
| SPEI03_trend | 0.0862 | 0.0000 | 0.1000 | 10 | 26.5 | 16.5 | 7 | 3 |
| SPEI12_mean | 0.2321 | 0.0000 | 0.2554 | 1 | 26.5 | 25.5 | 1 | 0 |
| SPEI12_trend | 0.1080 | 0.0000 | 0.1232 | 5 | 26.5 | 21.5 | 3 | 2 |
| SUN_mean | 0.1120 | 0.1615 | 0.1220 | 4 | 2 | 2 | 4 | 0 |
| SUN_trend | 0.0559 | 0.0949 | 0.0552 | 21 | 10 | 11 | 18 | 3 |
| TEM_mean | 0.0602 | 0.0989 | 0.0499 | 18 | 7 | 11 | 20 | 2 |
| TEM_trend | 0.1022 | 0.1070 | 0.1216 | 7 | 6 | 1 | 5 | 2 |
| WIND_mean | 0.0765 | 0.1362 | 0.0807 | 13 | 3 | 10 | 12 | 1 |
| WIND_trend | 0.0662 | 0.0930 | 0.0804 | 16 | 12 | 4 | 13 | 3 |

Table S3 Comparison between the proposed EEMD LightGBM SHAP framework and commonly used methods.

| Method | Main Use | Limitation | Advantage of This Study |
| --- | --- | --- | --- |
| OLS / Theil-Sen / MK | Detect monotonic trend | Cannot identify nonlinear turning points | EEMD separates nonlinear residual trend and identifies trend transition types |
| Correlation / Linear regression | Estimate average relationships | Assumes linear/additive effects | LightGBM captures nonlinear and interactive effects |
| RESTREND | Separates climate and residual effects | Sensitive to linear model assumptions | ML model handles complex factor combinations |
| RF/GBM feature importance | Ranks variables | Limited direction and threshold interpretation | SHAP gives class-specific contribution and response curves |
| GWR / GTWR | Spatially varying regression | Usually linear in local form | LightGBM SHAP better captures nonlinear response structures |
